# Distinguishing features of long COVID identified through immune profiling

**DOI:** 10.1038/s41586-023-06651-y

**Published:** 2023-09-25

**Authors:** Jon Klein, Jamie Wood, Jillian R. Jaycox, Rahul M. Dhodapkar, Peiwen Lu, Jeff R. Gehlhausen, Alexandra Tabachnikova, Kerrie Greene, Laura Tabacof, Amyn A. Malik, Valter Silva Monteiro, Julio Silva, Kathy Kamath, Minlu Zhang, Abhilash Dhal, Isabel M. Ott, Gabrielee Valle, Mario Peña-Hernández, Tianyang Mao, Bornali Bhattacharjee, Takehiro Takahashi, Carolina Lucas, Eric Song, Dayna McCarthy, Erica Breyman, Jenna Tosto-Mancuso, Yile Dai, Emily Perotti, Koray Akduman, Tiffany J. Tzeng, Lan Xu, Anna C. Geraghty, Michelle Monje, Inci Yildirim, John Shon, Ruslan Medzhitov, Denyse Lutchmansingh, Jennifer D. Possick, Naftali Kaminski, Saad B. Omer, Harlan M. Krumholz, Leying Guan, Charles S. Dela Cruz, David van Dijk, Aaron M. Ring, David Putrino, Akiko Iwasaki

**Affiliations:** 1 Department of Immunobiology, Yale School of Medicine, New Haven, CT USA; 2 Abilities Research Center, Icahn School of Medicine at Mount Sinai, New York, NY USA; 3 Department of Ophthalmology, USC Keck School of Medicine, Los Angeles, CA USA; 4 Department of Dermatology, Yale School of Medicine, New Haven, CT USA; 5 Yale Institute for Global Health, Yale School of Public Health, New Haven, CT USA; 6 SerImmune, Goleta, CA USA; 7 Department of Internal Medicine (Pulmonary, Critical Care and Sleep Medicine), Yale School of Medicine, New Haven, CT USA; 8 Department of Microbiology, Yale School of Medicine, New Haven, CT USA; 9 Center for Infection and Immunity, Yale School of Medicine, New Haven, CT USA; 10 Department of Neurology and Neurological Sciences, Stanford University, Palo Alto, CA USA; 11 Howard Hughes Medical Institute, Chevy Chase, MD USA; 12 Department of Pediatrics (Infectious Diseases), Yale New Haven Hospital, New Haven, CT USA; 13 Department of Epidemiology of Microbial Diseases, Yale School of Public Health, New Haven, CT USA; 14 Department of Internal Medicine (Infectious Diseases), Yale School of Medicine, New Haven, CT USA; 15 Center for Outcomes Research and Evaluation, Yale New Haven Hospital, New Haven, CT USA; 16 Section of Cardiovascular Medicine, Department of Internal Medicine, Yale School of Medicine, New Haven, CT USA; 17 Department of Health Policy and Management, Yale School of Public Health, New Haven, CT USA; 18 Department of Biostatistics, Yale School of Public Health, New Haven, CT USA; 19 Department of Computer Science, Yale University, New Haven, CT USA; 20 Department of Internal Medicine (Cardiology), Yale School of Medicine, New Haven, CT USA; 21 Department of Rehabilitation and Human Performance, Icahn School of Medicine at Mount Sinai, New York, NY USA

**Keywords:** Viral infection, Cytokines, Antibodies, SARS-CoV-2

## Abstract

Post-acute infection syndromes may develop after acute viral disease^1^. Infection with SARS-CoV-2 can result in the development of a post-acute infection syndrome known as long COVID. Individuals with long COVID frequently report unremitting fatigue, post-exertional malaise, and a variety of cognitive and autonomic dysfunctions^2–4^. However, the biological processes that are associated with the development and persistence of these symptoms are unclear. Here 275 individuals with or without long COVID were enrolled in a cross-sectional study that included multidimensional immune phenotyping and unbiased machine learning methods to identify biological features associated with long COVID. Marked differences were noted in circulating myeloid and lymphocyte populations relative to the matched controls, as well as evidence of exaggerated humoral responses directed against SARS-CoV-2 among participants with long COVID. Furthermore, higher antibody responses directed against non-SARS-CoV-2 viral pathogens were observed among individuals with long COVID, particularly Epstein–Barr virus. Levels of soluble immune mediators and hormones varied among groups, with cortisol levels being lower among participants with long COVID. Integration of immune phenotyping data into unbiased machine learning models identified the key features that are most strongly associated with long COVID status. Collectively, these findings may help to guide future studies into the pathobiology of long COVID and help with developing relevant biomarkers.

## Abstract (web summary)

Individuals with long COVID show marked biological changes in cortisol and immune factors relative to convalescent populations.

## Main

Recovery from acute viral infections is heterogeneous and chronic symptoms may linger for months to years in some individuals. Moreover, persistent sequelae may develop after acute infection by a number of viruses from a diverse range of viral families^5–9^. Post-acute infection syndromes (PAIS) following microbial infections have also been described for over a century^10,11^. Yet despite their ubiquity, the basic biology underlying PAIS development, even for extensively studied PAIS such as myalgic encephalomyelitis/chronic fatigue syndrome, remains unclear^1,12^.

SARS-CoV-2 is a *Betacoronavirus* that is responsible for almost 7 million deaths worldwide^13^. Infection causes COVID-19, which can manifest as a severe respiratory disease marked by extensive immunological and multiorgan system dysfunction^14–19^. Recovery from COVID-19 is often complete; however, individuals (even those with initially mild disease courses) may have increased risks for adverse clinical events and abnormal clinical findings^20–25^.

In addition to developing isolated dysfunctions, some patients recovering from COVID-19 may develop a group of new onset or aggravated sequelae known as long COVID (LC). Clinically, LC presents as a constellation of debilitating symptoms including unremitting fatigue, post-exertional malaise, cognitive impairment and autonomic dysfunction, alongside other less common manifestations^2–4^. These persistent sequelae markedly impair physical and cognitive function and reduce quality of life^26^. Estimates of LC prevalence vary substantially^27^, but prospective studies suggest that about one in eight individuals with COVID-19 experience persistent somatic symptoms that are attributable to past SARS-CoV-2 infection^28^. Although the underlying pathogenesis of LC remains unclear, current hypotheses include the persistence of virus or viral remnants in tissues; development or aggravation of autoimmunity; microbial dysbiosis; reactivation of non-SARS-CoV-2 latent viral infections; and tissue damage caused by chronic inflammation.

To investigate the biological underpinnings of LC, a cross-sectional study was designed (Mount Sinai–Yale long COVID; hereafter, MY-LC) involving 275 participants comprising five study groups: (1) healthcare workers infected with SARS-CoV-2 before vaccination (HCW); (2) healthy, uninfected, vaccinated controls (healthy control (HC) group); (3) previously infected, vaccinated controls without persistent symptoms (convalescent control (CCs) group); (4) individuals with persistent symptoms after acute infection (LC); and (5) a second group of individuals with persistent symptoms after acute infection from an independent study (external LC, hereafter EXT-LC). Among the CC and LC groups, enrolled participants had primarily mild (non-hospitalized) acute COVID-19 and samples for this study were acquired, on average, more than a year after their acute infection. The HC, CC and LC groups underwent systematic, multidimensional immunophenotyping and unbiased machine learning of aggregated data to identify potential LC biomarkers.

## Overview of the MY-LC cohort

**Fig. 1:**
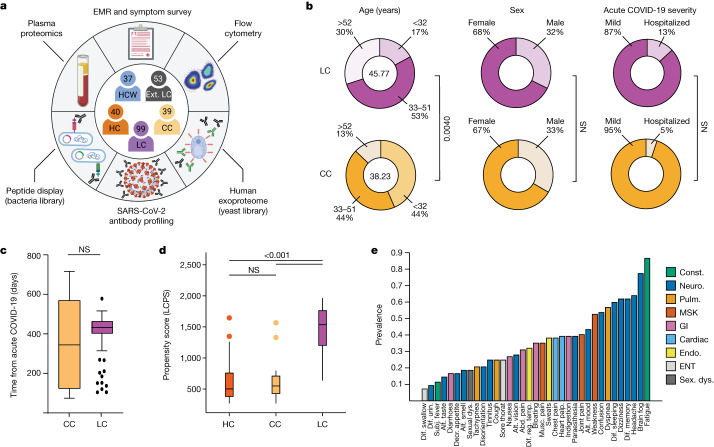
Demographic and clinical stratification of participants with LC. **a**, Schematic of the MY-LC study. Numbers indicate the number of participants after exclusion (Methods). The diagram was created using BioRender. **b**, Select demographic information for the LC (top row, purple) and CC (bottom row, yellow) groups. The centre values in the ‘age’ column represent the average group values. *n* = 39 (CC) and *n* = 99 (LC). Statistical significance is reported for relevant post hoc comparisons (age) or *χ*^2^ tests (sex and acute disease severity). Complete statistical results are shown in Extended Data Table 1. **c**, The time (days) from acute symptom onset between the LC and CC groups. Significance was assessed using a two-tailed Brown–Mood median test with an alpha of 0.05. NS, not significant. *n* = 39 (CC) and *n* = 99 (LC). **d**, The LCPS for each individual. *n* = 40 (HC), *n* = 39 (CC) and *n* = 98 (LC). Significance was assessed using Kruskal–Wallis tests corrected for multiple comparisons using the Bonferroni method. **e**, The prevalence of the top 30 self-reported binary symptoms ranked from most prevalent (right) to least prevalent (left). Symptoms are coloured according to common physiological system: constitutional (const., green), neurological (neuro., dark blue), pulmonary (pulm., gold), musculoskeletal (MSK, red), gastrointestinal (GI, pink), cardiac (light blue), endocrine (endo., yellow), ear, nose and throat (ENT, light grey), and sexual dysfunction (sex. dys., dark grey). For the box plots in **c** and **d**, the central lines indicate the group median values, the top and bottom lines indicate the 75th and 25th percentiles, respectively, the whiskers represent 1.5× the interquartile range and individual datapoints mark outliers. abd., abdominal; alt., altered; decr., decreased; dif., difficulty; EMR, electronic medical record; IQR, interquartile range; musc., muscle; palp., palpitations; reg., regulating; subj., subjective; temp., body temperature; Urin., urination.

The MY-LC study enrolled 185 participants (101 LC, 42 CC and 42 HC) at one study site (Mount Sinai Hospital) and 90 participants at another (Yale New Haven Hospital) for a total of 275 participants. After initial enrolment and preliminary review of electronic medical records, two participants were excluded from the LC group (2.0%, for pharmacological immunosuppression secondary to primary immune deficiency and solid organ transplant); two from the HC group (4.8%, for pregnancy and misclassification at enrolment); and three from the CC group (7.1%, for pregnancy, monogenic disorder and misclassification at enrolment) resulting in a final study size of 268 individuals (Fig. 1a). The proportion of participants excluded from the LC group did not significantly differ from those excluded from the other groups (Extended Data Table 1).

Initial comparison of demographic factors showed the LC and CC groups differed in mean age (46 years, LC; 38 years, CC; Kruskal–Wallis with post hoc Bonferroni correction, *P* = 0.0040). However, these groups did not significantly differ in sex, hospitalization for acute COVID-19 (Fig. 1b) or median elapsed time between initial infection and acute disease (Fig. 1c). Most acute infections within the LC group (76%) occurred between epidemiological weeks 7–17 of 2020, when parental SARS-CoV-2 strains (WA-1) drove the majority of new cases. Importantly, the aggregated medical history of individuals with LC did not significantly differ from that of CC individuals in prevalence of anxiety or depression. Complete demographic features and medical histories are reported in Extended Data Table 1.

**Extended Data Fig. 1:**
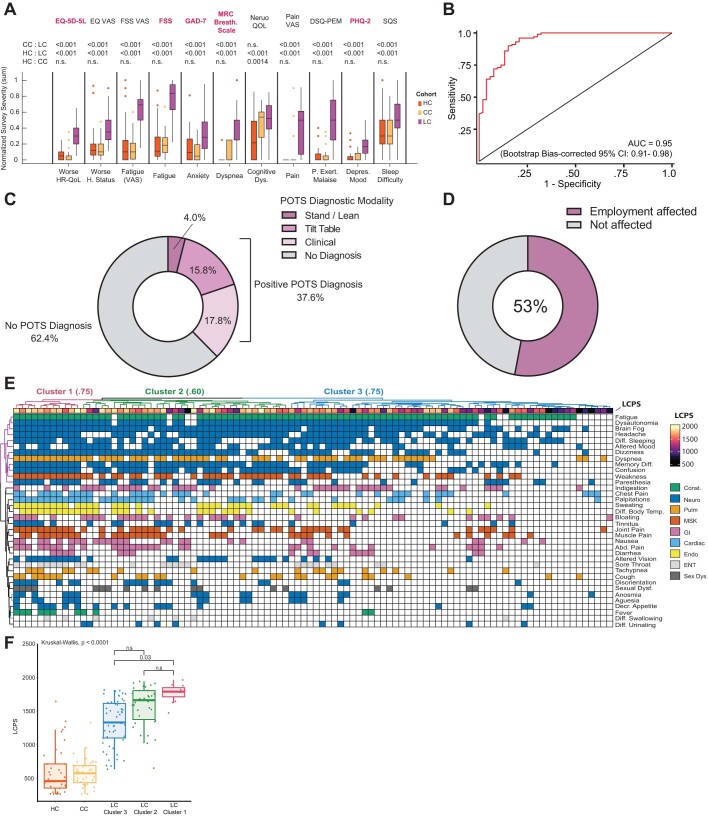
Additional demographic and clinical analysis of Long COVID cohort. (**A**) Box plots of Min-Max normalized survey responses (n = 40 HC, 38 CC, 91 LC). Only participants who completed all surveys were included. Individual survey instruments are arranged in columns with corresponding health dimensions below. Surveys in red were aggregated to generate Long COVID Propensity Scores (LCPS). Significance was assessed using Kruskal-Wallis tests corrected for multiple comparisons using Bonferroni’s method. (**B**) Receiver-Operator Curve (ROC) analysis of LCPS scores. Area under the curve (AUC) is reported with Bootstrap Bias-corrected 95% confidence intervals (CI) of AUC. (**C**) Ring plots of prevalence of Postural Orthostatic Tachycardia Syndrome (POTS) among Long COVID cohort (n = 99). “No diagnosis” is represented by grey regions, “positive diagnosis” is represented by shaded purple regions. Purple regions are further stratified by diagnostic modality: clinical = diagnosed through clinical evaluation (light purple); Tilt-table = diagnosed by Tilt-table (middle purple); Stand / Lean = diagnosed by Stand / LEAN test (dark purple). (**D**) Ring plots of prevalence of self-reported negative impacts on employments status among individuals with Long COVID (n = 99). Negative responses are represented by grey region, positive responses are indicated by purple region. (**E**) Heatmap of self-reported binary symptoms clustered by Hamming distances (rows and columns) and coloured according to physiological system as previous. Columns are annotated by LCPS scores with bootstrapped cluster reproducibility scores reported in parentheses (bootstrapped Jaccard similarity) (**F**) Boxplots of Long Covid Propensity Score (LCPS) plotted by group (HC = healthy control; CC = convalescent control; LC = Long COVID) and cluster. Central lines represent group medians, bottom and top lines represent 25^th^ and 75^th^ percentiles, respectively. Whiskers represent 1.5× inter-quartile range (IQR). Significance for difference in median LCPS was assessed using Kruskal-Wallis with correction for multiple comparisons using Bonferroni-Holm.

Across all surveyed dimensions, participants with LC had significantly higher intensities of reported symptoms and a substantially worsened quality of life (Extended Data Table 2 and Extended Data Fig. 1a). To address whether LC associated with any pattern of survey responses, responses were aggregated into a single classification metric (LC propensity score (LCPS)) using a parsimonious logistic regression model (LC versus other), which demonstrated significant diagnostic potential (area under the curve (AUC) = 0.95, bootstrap 95% confidence interval (CI) = 0.91–0.98; Fig. 1d, Extended Data Fig. 1b and Extended Data Table 3).

Among the self-reported symptoms from the LC group, fatigue (87%), brain fog (78%), memory difficulty (62%) and confusion (55%) were most common (Fig. 1e). Postural orthostatic tachycardia syndrome (POTS) was also prevalent; 38% of individuals with LC had formal diagnostic testing and clinical evaluation (Extended Data Fig. 1c). Negative impacts on employment status were also reported by half of the participants with LC (Extended Data Fig. 1d).

To find groups of participants with LC with similar sets of self-reported symptoms, an agglomerative hierarchical clustering of binary symptoms was performed (Extended Data Fig. 1e). Three LC clusters were identified (bootstrapped mean cluster-wise Jaccard similarity: cluster 1, 0.75 (95% CI = 0.54–1.00); cluster 2, 0.60 (95% CI = 0.47–0.94); and cluster 3, 0.75 (95% CI = 0.56–1.00)). LC clusters were bifurcated by LCPS: cluster 3 had intermediate propensity scores; clusters 1 and 2 had more extreme scores (Extended Data Fig. 1f).

## Differences in circulating immune cells

**Extended Data Fig. 2:**
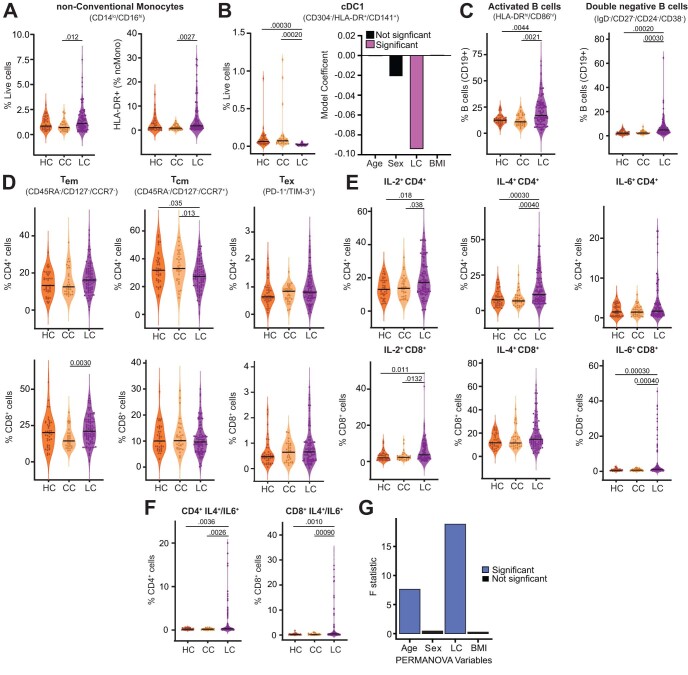
Immunological differences in myeloid and lymphocyte effectors among participants with Long COVID. (**A-B**) Violin plots of myeloid peripheral blood mononuclear populations (PBMCs) plotted by group as percentages of respective parent populations (gating schemes detailed in Extended Data Fig. 10). **(B**, **right)** Coefficients from linear model are shown. Model predictors are indicated on x-axis. Significant predictors (p ≤ 0.05) are plotted in purple. Detailed model results are reported in Extended Data Table 4. (**C**) Violin plots of B lymphocyte subsets from PBMCs plotted as percentages of respective parent populations (gating schemes detailed in Extended Data Fig. 10). (**D**,**E**) Violin plots of various CD4^+^ (top row) and CD8^+^ (bottom row) populations. (**F**) Violin plots of IL-4 and IL-6 double-positive CD4^+^ (left) and CD8^+^ (right) T cells plotted as percentages of total CD4^+^ or CD8^+^ T cells. (**G**) A PERMANOVA test of the association between all cell populations shown and participant age, sex, LC status, and body mass index (BMI). For all violin plots (**A**–**F**), significance was assessed using Kruskal-Wallis corrected for multiple comparisons using Bonferroni-Holm. Each dot represents a single patient (n = 40 HC, 33 CC, 99 LC). Central bars indicate the median value of each group. Only significant differences between group medians are shown.

**Extended Data Fig. 3:**
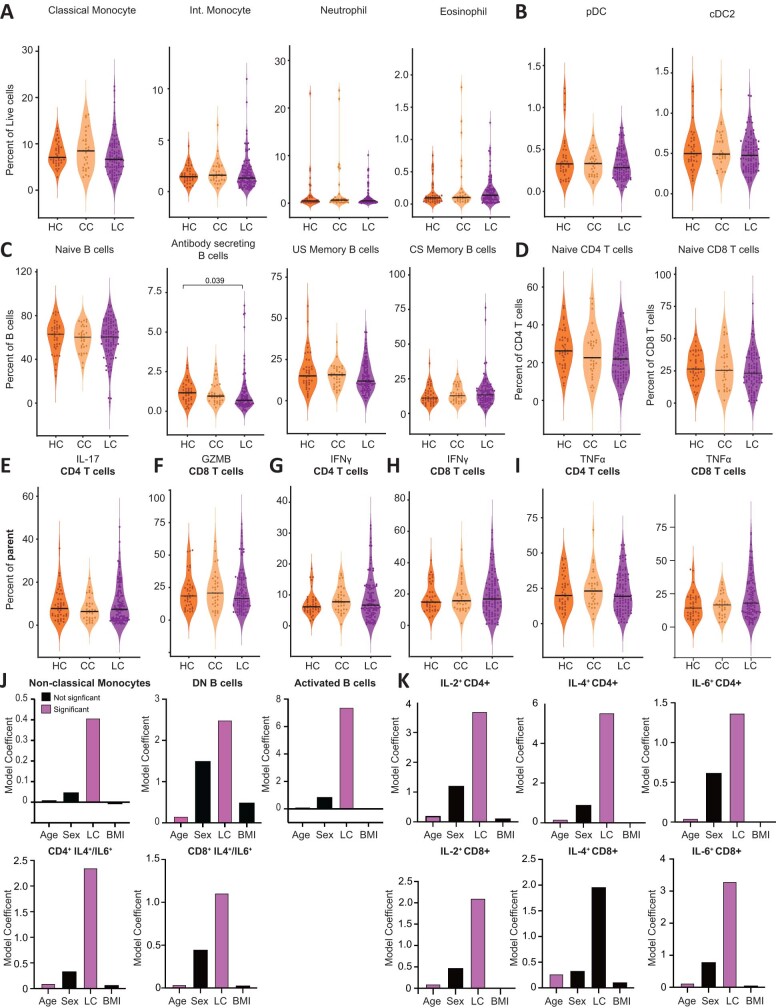
Circulating myeloid, B cell, and cytokine producing immune cell populations among MY-LC participants. (**A**–**I**) Violin plots of various myeloid, B, and T cell PBMC populations stratified by healthy (HC), convalescent (CC), and Long COVID (LC) groups. Significance for differences in group medians was assessed using Kruskal-Wallis tests with correction for multiple comparisons using Bonferroni-Holm. Each dot represents a single patient (n = 40 HC, 33 CC, 99 LC) (**J**–**K**) Coefficients from linear models for various PBMC populations. Bars in purple indicate significant predictors of specific PBMC populations (p ≤ 0.05).

**Extended Data Fig. 4:**
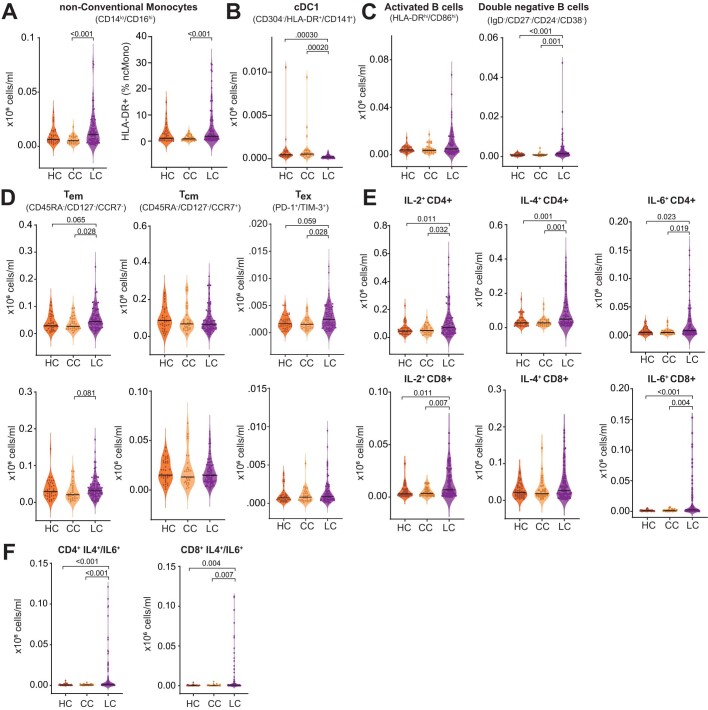
Absolute Counts of in myeloid and lymphocyte effectors among participants with Long COVID. (**A**-**B**) Violin plots of myeloid peripheral blood mononuclear populations (PBMCs) plotted by group (HC, healthy control; CC, convalescent control; LC, Long COVID) as absolute cell counts (gating schemes detailed in Extended Data Fig. 10a). Significance for differences in group medians was assessed using Kruskal-Wallis tests with correction for multiple comparisons using Bonferroni-Holm. (**C**) Violin plots of B lymphocyte subsets from peripheral blood mononuclear populations (PBMCs) plotted as absolute cell counts (gating schemes detailed in Extended Data Fig. 10d). Significance was assessed using Kruskal-Wallis with correction for multiple comparison using Bonferroni-Holm. (**D**, **E**) Violin plots of various CD4 (top row) and CD8 (bottom row) populations. Significance was assessed using Kruskal-Wallis with correction for multiple comparison using Bonferroni-Holm. (**F**) Violin plots of IL-4 and IL-6 double positive CD4^+^ (left) and CD8^+^ (right) T cells plotted as absolute cell counts. Significance was assessed using Kruskal-Wallis with correction for multiple comparison using Bonferroni-Holm. For all plots (**A**–**F**), central bar in the violin plot indicated the median value of each group. Each dot represents a single patient (n = 37 HC, 28 CC, 94 LC).

Analysis of peripheral blood mononuclear cell (PBMC) populations revealed a significant difference in circulating immune cell populations among the MY-LC cohorts. The median level of non-conventional monocytes (CD14^low^CD16^high^) in the LC group was significantly higher compared with the levels in the CC group (Extended Data Fig. 2a (left)). To determine whether LC is significantly associated with levels of non-conventional monocytes after accounting for demographic differences across all groups, linear models were developed incorporating age, sex, LC status (binary) and body mass index (BMI). Using this approach, LC was significantly associated with levels of total non-conventional monocytes (Extended Data Fig. 3j). Expression of MHC class II (HLA-DR) was also significantly elevated in LC relative to the CC group (Extended Data Fig. 2a (right)). Parallel investigation of absolute cell counts revealed similar increases (Extended Data Fig. 4a).

Systematic analysis of other immune effector populations revealed significantly lower circulating populations of conventional type 1 dendritic (cDC1) cells among participants with LC (Extended Data Figs. 2b (left) and 4b). Linear models again found that LC status and age were significantly associated with circulating cDC1 levels (Extended Data Fig. 2b (right)). The levels of other circulating granulocyte populations (neutrophils, eosinophils, conventional and intermediate monocytes, plasmacytoid dendritic and cDC2 populations) did not significantly differ among groups, with substantial heterogeneities noted in LC (Extended Data Fig. 3a,b).

The median relative percentage of B lymphocytes was significantly higher in both activated populations (CD86^high^HLA-DR^high^: 17% (LC), 11% (CC) and 12% (HC)) and double-negative subsets (IgD^–^CD27^–^CD24^–^CD38^–^: 5% (LC), 2% (CC) and 2% (HC)) (Extended Data Fig. 2c). The absolute count of double-negative B cells also significantly increased in individuals with LC (Extended Data Fig. 4c). LC status was again significantly associated with these effector populations in linear modelling (Extended Data Fig. 3j). Circulating levels of other B cell subsets, including naive B cells, did not significantly differ among groups (Extended Data Fig. 3c).

Circulating T lymphocyte populations were not notably different in effector memory subsets (CD45RA^–^CD127^–^CCR7^–^) (Extended Data Fig. 2d), although absolute counts of CD4^+^ populations significantly increased (Extended Data Fig. 4d). The median relative percentage of circulating CD4^+^ central memory cells (CD45RA^–^CD127^+^CCR7^–^) was significantly lower in the LC group (27% (LC), 33% (CC) and 32% (HC)), although the groups did not differ by absolute counts (Extended Data Fig. 4d). Median percentages of exhausted (PD-1^+^TIM3^+^) CD4^+^ subsets and exhausted CD8^+^ subsets did not significantly differ (Extended Data Fig. 2d), but absolute exhausted CD4^+^ T cell counts were significantly elevated (Extended Data Fig. 4d). Importantly, neither naive CD4^+^ nor CD8^+^ T cells significantly differed (Extended Data Fig. 3d).

After being stimulated with phorbol myristate acetate and ionomycin, CD4^+^ cells from individuals with LC produced significantly higher median levels of intracellular IL-2 (17% (LC), 14% (CC) and 13% (HC)) and IL-4 (11% (LC), 7% (CC) and 8% (HC)) (Extended Data Figs. 2e and 4e (top row)), as well as IL-2 (4% (LC), 2% (CC), 2% (HC)) and IL-6 (1.2% (LC), 0.6% (CC), 0.6% (HC)) among CD8^+^ T cells (Extended Data Figs. 2e and 4e (bottom row)). Both age and LC status were significantly associated with intracellular IL-2 (CD4^+^/CD8^+^), IL-4 (CD4^+^) and IL-6 (CD8^+^) production (Extended Data Fig. 2k and Extended Data Table 4). Notably, individuals with LC also had uniquely elevated median levels of IL-4/IL-6 double-positive CD4^+^ T cells (0.3% (LC), 0.2% (CC) and 0.2% (HC)) and IL-4/IL-6 double-positive CD8^+^ T cells (0.5% (LC), 0.2% (CC) and 0.2% (HC)) (Extended Data Figs. 2f and 4f). The levels of IFNγ and IL-17 (in CD4^+^ cells) and TNF and GMZB (in CD8^+^ cells) did not significantly differ across groups (Extended Data Fig. 3e–i). To account for heterogeneous levels of circulating immune cell populations, permutational analysis of variance (PERMANOVA) was performed using effector populations with significant differences between groups at the baseline. This multivariate analysis showed that LC status and age significantly predicted levels of circulating immune cell populations (Extended Data Fig. 2g).

## SARS-CoV-2-specific antibody responses

**Fig. 2:**
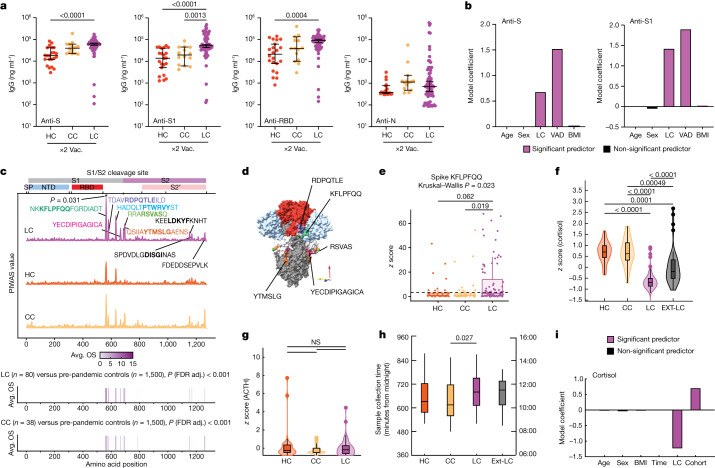
Exaggerated SARS-CoV-2-specific humoral responses and altered circulating immune mediators among participants with LC. **a**, The SARS-CoV-2 antibody responses were assessed using ELISA. *n* = 22 (HC), *n* = 14 (CC) and *n* = 69 (LC). The vaccination (vac.) status for each cohort is indicated (×2), indicating the number of SARS-CoV-2 vaccine doses at sample collection. Significance for difference in group median values was assessed using Kruskal–Wallis with Benjamini–Hochberg false-discovery rate (FDR) correction for multiple comparisons. The central lines indicate the group median values and the whiskers show the 95% CI estimates. **b**, Coefficients from linear models are reported. Model predictors are indicated on the *x* axis. Significant predictors (*P* ≤ 0.05) are shown in purple. Detailed model results are shown in Extended Data Table 5. **c**, PIWAS line profiles of IgG binding within participants with more than 1 vaccine dose plotted along the SARS-CoV-2 spike amino acid sequence. Various spike protein domains are indicated by coloured boxes (top). 95th percentile values are arranged by group: LC (purple, *n* = 80), HC (orange, *n* = 39) and CC (yellow, *n* = 38); peaks with a PIWAS value of ≥2.5 are annotated by their consensus linear motif sequence (bold) and surrounding residues. Significantly enriched peaks in the LC group are indicated by an asterisk (*), as calculated using outlier sum (OS) statistics. **d**, Three-dimensional mapping of LC-enriched motif sequences onto trimeric spike protein. Light grey, S1; light blue, N-terminal domain; red, RBD; dark grey, S2. Various LC-enriched motifs are annotated. **e**, *z*-score enrichments for IgG binding to the spike sequence KFLPFQQ among participants who have received at least one vaccine dose. A *z* score of >3 indicates significant binding relative to the control populations. **f**–**h**, *z*-score-transformed cortisol (**f**) ACTH (**g**) and sample-collection times (**h**) by group. Participants with potentially confounding medical comorbidities (such as pre-existing pituitary adenoma, adrenal insufficiency and recent oral steroid use) were removed before analysis. *n* = 39 (HC), *n* = 39 (CC), *n* = 93 (LC). **i**, Coefficients from linear models of cortisol levels. Significant predictors (*P* ≤ 0.05) are shown in purple. Detailed model results are reported in Extended Data Table 6. For the box plots in **e**–**h**, the central lines indicate the group median values, the top and bottom lines indicate the 75th and 25th percentiles, respectively, the whiskers represent 1.5× the interquartile range and individual datapoints mark outliers. Significance for differences in group median values was assessed using Kruskal–Wallis tests with Bonferroni’s correction for multiple comparisons. SP, signal peptide.

**Extended Data Fig. 5:**
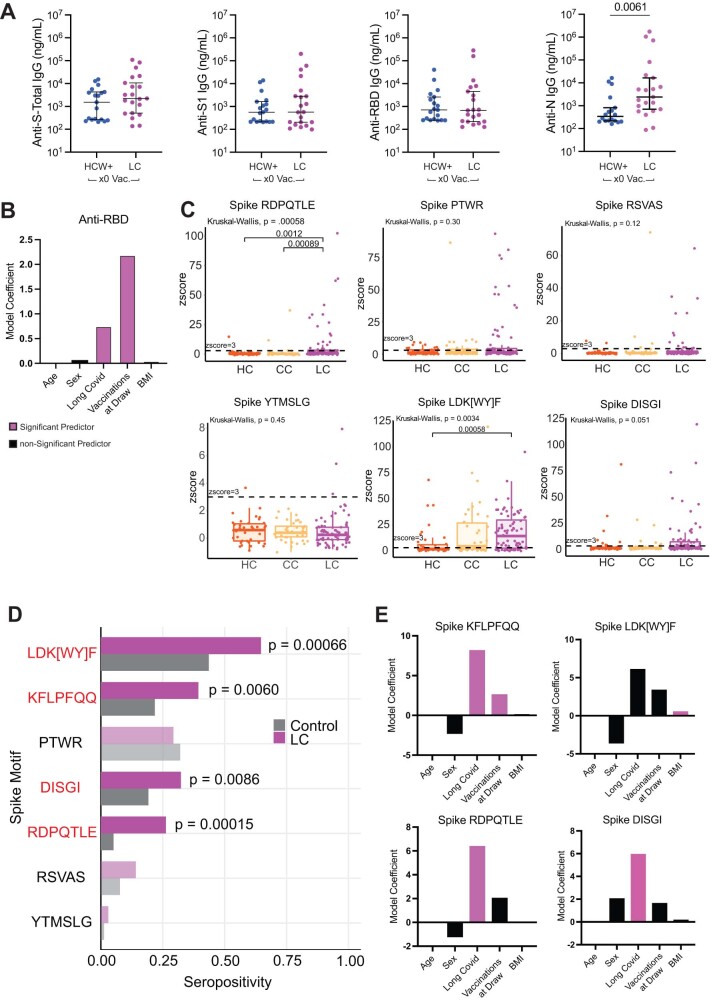
Humoral Analysis of SARS-CoV-2 specific antibodies. (**A**) Dot plots of IgG concentrations from historical, unvaccinated SARS-CoV-2 exposed controls (HCW+) and unvaccinated Long COVID participants. Central lines indicate median group values with bars representing 95% CI estimates. Vaccination status for each cohort is indicated by the form “x0” where the digit indicates the number of SARS-CoV-2 vaccine doses. Significance for differences in group medians were assessed using the Mann-Whitney test. Each dot represents a single patient (n = 19 HCW, 19 LC). (**B**) Coefficients from linear models are reported for anti-RBD antibody responses. Model predictors are reported along the x-axis and included age, sex (categorical), Long COVID status (categorical), body mass index (BMI), and number of vaccinations at blood draw. Significant predictors (p ≤ 0.05) are plotted in purple. Detailed model results are reported in Extended Data Table 5. (**C**) Boxplots of antibody binding to various SARS-CoV-2 linear peptide sequences plotted by group (HC = healthy control; CC = convalescent control; LC = Long COVID) amongst participants who have received 1 or more vaccine doses. Each dot represents one individual. Central bars represent groups medians, with bottom and top bars representing 25^th^ and 75^th^ percentiles, respectively. Dashed line represents z-score threshold for epitope positivity defined by SERA. Statistical significance determined by Kruskal-Wallis with correction for multiple comparisons using Bonferroni-Holm. Each dot represents an individual patient: LC (purple, n = 80), HC (orange, n = 39) and CC (yellow, n = 38). (**D**) Proportion of each group amongst participants who have received 1 or more vaccine doses (LC: n = 80, control: n = 77) that is seropositive (z-score ≥ 3) for each of 7 linear Spike motifs mapping to outlier peaks. Motifs with significantly different seropositivity between groups are highlighted in red, as determined by Fisher’s exact test corrected for multiple comparisons by FDR (Benjamini-Hochberg). (**E**) Coefficients from linear models are reported for anti-RBD antibody responses. Model predictors are reported along the x-axis and included age, sex (categorical), Long COVID status (categorical), body mass index (BMI), and number of vaccinations at blood draw. Significant predictors (p ≤ 0.05) are plotted in purple. Detailed model results are reported in Extended Data Table 5. *Abbreviation: HCW+, previously SARS-CoV-2 infected healthcare worker*.

Initial analysis of anti-SARS-CoV-2 antibody responses was performed only for participants in the MY-LC group who received two doses of vaccine. Anti-S1 IgG levels in the LC group were significantly higher compared with those in the CC group, and the levels of total anti-S and anti-receptor-binding domain (RBD) IgG were elevated in the LC group but did not significantly differ from the levels in the CC group (Fig. 2a). Unvaccinated participants with LC had significantly higher anti-N IgG levels compared against a subset of historical, unvaccinated controls who were previously infected with SARS-CoV-2 (Extended Data Fig. 5a).

Linear models were constructed to more fully account for baseline differences (demographics, vaccines at blood draw (VAD)) across cohorts (Fig. 2b and Extended Data Fig. 5b), which revealed that LC state was a significant, positive predictor of anti-spike humoral response after accounting for such differences (Extended Data Table 5). To gauge whether the elevated responses were to distinct regions of spike, anti-SARS-CoV-2 IgG responses against linear peptides were profiled among vaccinated participants. The responses of participants with LC were significantly greater than CC responses against a peptide that confers increased neutralization^29,30^, corresponding to amino acid residues 556–572 (1.3×; outlier sum, *P* = 0.031). Responses were also greater (1.4×–1.6×) for peptides corresponding to residues 572–586, 625–638 and 682–690 (the furin-cleavage site). CC participant responses were higher than the LC group responses against two S2 peptides (residues 1149–1161, 1.5×; 1256–1266, 2.1×) (Fig. 2c). Multiple differentially expressed spike-binding motifs were mapped onto available trimeric-structure models of spike (Protein Data Bank (PDB): 6VXX). These mapped to highly surface exposed sites in the protein’s natural conformational state, near the S1 RBD (RDPQTLE and KFLPQQ) and the S1/S2 cleavage site (RSVAS, YECDIPIGAGICA and YMSLG) (Fig. 2d), consistent with participants with LC having higher anti-spike immune responses. By analysing peptide enrichment for spike motifs corresponding to peaks identified in a protein-based immunome-wide association study (PIWAS), significantly greater humoral responses against KFLPFQQ (Kruskal–Wallis, *P* = 0.023) (Fig. 2e), RDPQTLE (*P* = 0.00058) and LDK[WY]F (*P* = 0.0034) were found (Extended Data Fig. 5c). Prevalences of antibody reactivities against KFLPFQQ (Fisher’s exact, *P* = 0.0060), RDPQTLE (*P* = 0.00015), LDK[WY]F (*P* = 0.00066) and DISGI (*P* = 0.0086) were also significantly higher among participants with LC than among grouped controls (Extended Data Fig. 5d). Statistical modelling accounting for baseline differences (demographics, VAD) revealed that LC is significantly associated with reactivity against KFLPFQQ, RDPQTLE and DISGI motifs (Extended Data Fig. 5e), but not with reactivity against LDK[WY]F (Extended Data Fig. 5e), which was elevated in both the CC and LC groups (Extended Data Fig. 5c).

## Cortisol and soluble immune mediators

**Extended Data Fig. 6:**
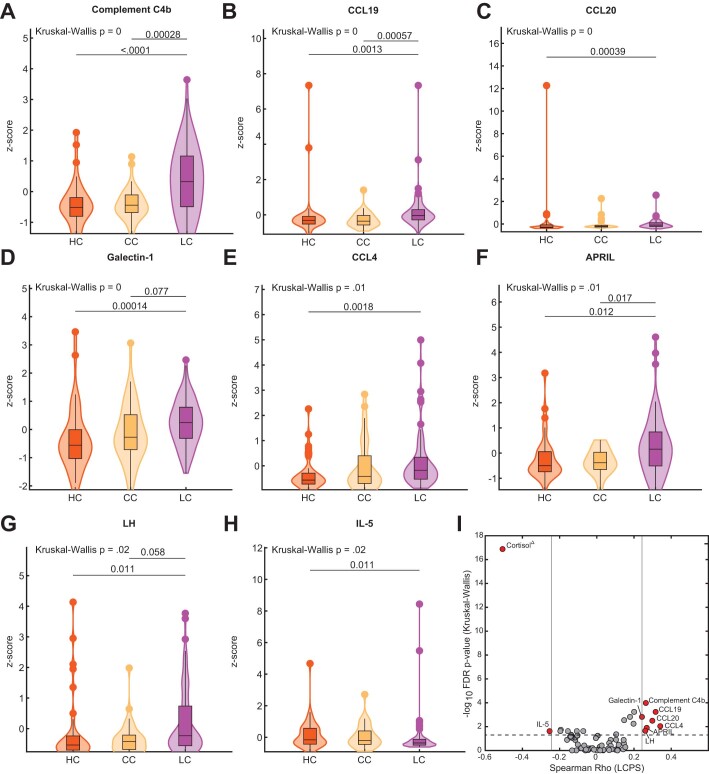
Significantly different soluble plasma factors across MY-LC cohorts. (**A**–**H**) Violin plots of various z-score transformed circulating plasma factors across healthy (HC), convalescent (CC), and Long COVID (LC) cohorts. Significance of difference in group medians was assessed using Kruskal-Wallis corrected for multiple comparisons using Bonferroni’s method. P-values from multiple Kruskal-Wallis testing were adjusted using the Benjamini-Hochberg procedure. (**I**) Negative Log_10_ transformed p-values from Kruskal-Wallis tests plotted against Spearman correlations with LCPS for various plasma factors. Reported p-values are adjusted for multiple comparisons using FDR (Benjamini-Hochberg). Horizontal line represents significance threshold for a difference in group medians. Vertical lines represent the minimum correlation values for plasma factors significantly correlating with LCPS scores. Red depicts factors with significant differences in group medians and significant correlations with LCPS.

Parallel multiplex analysis of circulating hormones and immune mediators in plasma samples revealed that the groups in the MY-LC cohort significantly differed in median levels of cortisol (Kruskal–Wallis, *P *< 0.0001), complement C4b (*P* = 0.0001), CCL19 (*P* = 0.00058), galectin-1 (*P* = 0.0015), CCL20 (*P* = 0.0032), CCL4 (*P* = 0.0092), APRIL (*P* = 0.013), LH (*P* = 0.022) and IL-5 (*P* = 0.024). Post hoc comparisons showed that the LC group had significantly increased complement C4b, CCL19, CCL20, galectin-1, CCL4, APRIL and LH, and marginally but significantly decreased IL-5 (Extended Data Fig. 6a–h). Additional analysis revealed significant correlations with LCPS scores, particularly for cortisol (Extended Data Fig. 6i). In the EXT-LC cohort (*n* = 53, excluding an outlier whose level was >8 s.d. above the median), cortisol levels in the LC group were lower than those in the HC and CC groups (Fig. 2f). Paired levels of adrenocorticotropic hormone (ACTH) were evaluated only in the MY-LC cohort; these did not significantly differ across groups (Fig. 2g). Median sample collection times significantly differed only between the CC and LC groups, and this difference was modest (65 min; Dunn’s test, *P* = 0.027) (Fig. 2h). Subsequent statistical modelling revealed that LC status significantly associated with lower cortisol levels after accounting for individual differences in age, sex, BMI, sample-collection time and cohort (MY-LC versus EXT-LC) (Fig. 2i and Extended Data Table 6).

## Autoantibodies to exoproteome

**Fig. 3:**
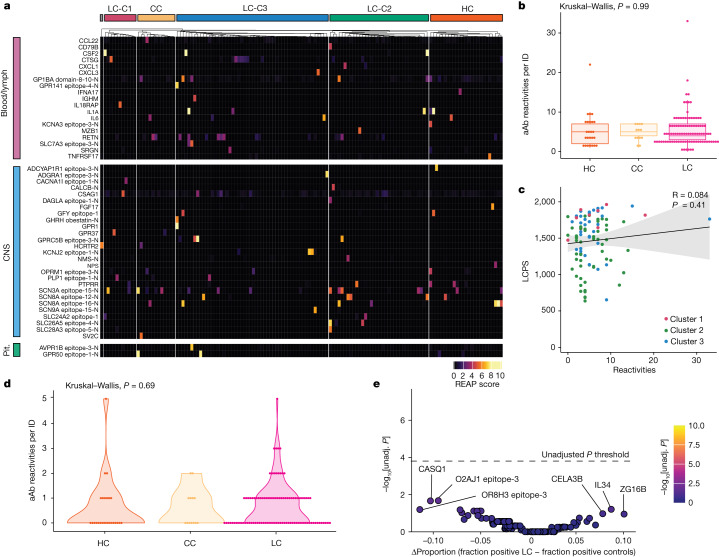
Participants with LC showed limited but selective autoantibodies against the human exoproteome. **a**, REAP reactivities across the MY-LC cohort. *n* = 25 (HC), *n* = 13 (CC) and *n* = 98 (LC). Each column is one participant, grouped by cohort (for HC and CC) or by LCPS (for LC). Column clustering within groups was performed by *k*-means clustering. Each row represents one protein. Proteins were grouped using Human Protein Atlas mRNA expression data for different tissues. Reactivities shown have at least one participant with a REAP score ≥3. Only reactivities enriched in blood/lymph, CNS or pituitary are shown for brevity. **b**, The number of autoantibody (aAb) reactivities per individual (ID) by group. Significance was assessed using Kruskal–Wallis tests. For the box plots, the central lines indicate the group median values, the top and bottom lines indicate the 75th and 25th percentiles, respectively, the whiskers represent 1.5× the interquartile range. Each dot represents one individual. **c**, The relationship between number of autoantibody reactivities per individual and LCPS. Correlation was assessed using Spearman’s correlation. The black line shows the linear regression, and the shading shows the 95% CIs. Colours show the LC LCPS group (red, cluster 1; green, cluster 2; blue, cluster 3). Each dot represents one individual. **d**, The number of GPCR autoantibodies per individual. Significance was assessed using Kruskal–Wallis tests. Each dot represents one individual. **e**, Assessment of the frequency of individual autoantibody reactivities in participants with LC and control individuals. Significance was assessed using Fisher’s exact tests. The *y* axis shows −log_10_-transformed unadjusted *P* values; the Bonferroni-adjusted significance threshold is indicated by a black dashed line. The *x* axis shows the difference in the proportion of autoantibody-positive individuals in each group. Each dot represents one autoantibody reactivity. CNS, central nervous system; pit., pituitary.

**Extended Data Fig. 7:**
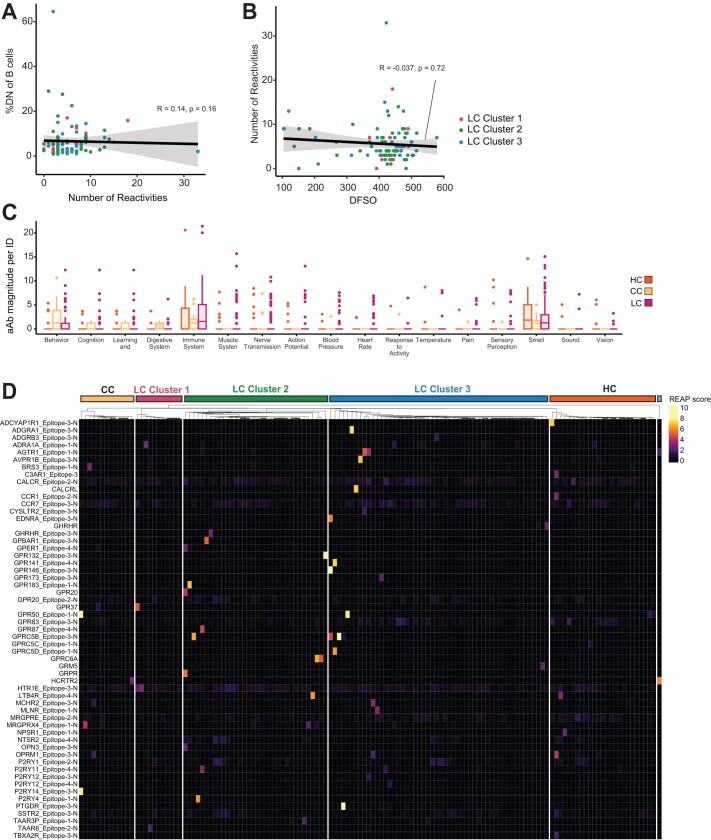
Analysis of private autoantibodies within the MY-LC cohort. (**A**–**B**) Correlation plots depicting relationships between number of autoantibody reactivities and %DN of B cells (**A**) or days from symptom onset (DFSO) and number of autoantibody reactivities (**B**). For all panels, correlation was assessed using Spearman’s method. Black line depicts linear regression with 95% CI shaded. Colours depict Long COVID cluster (cluster 3, blue; cluster 2, green; cluster 1, red). Each dot represents one individual. (**C**) Grouped box plot depicting reactivity magnitude per individual in the listed GO Process domain. Reactivity magnitude is calculated as the sum of REAP scores for all reactivities per individual in a given GO Process domain. Statistical significance assessed by Kruskal-Wallis and adjusted for multiple comparisons using FDR (Benjamini-Hochberg) correction. Boxplot coloured box depicts 25th to 75th percentile of the data, with the middle line representing the median, the whiskers representing 1.5× the interquartile range, and outliers depicted as points. (**D**) Heatmap depicting autoantibody reactivity for GPCRs included in the REAP library. Each column is one participant, grouped by control or LCPS cluster. HC = healthy control, CC = convalescent control, LC = Long COVID. *Abbreviations: GPCR = G-protein coupled receptor*.

Next, antibody reactivity against extracellular proteins was assessed in 98 participants with LC and 38 control participants using rapid extracellular antigen profiling (REAP)—a method used to measure antibody reactivity against more than 6,000 extracellular and secreted human proteins^16^. Although participants with LC had a variety of private reactivities against diverse autoantigens (Fig. 3a), the number of autoantibody reactivities per participant did not differ across groups (Fig. 3b), nor did the number of reactivities significantly correlate with LC clusters (as assessed by LCPS scores) (Fig. 3c). Moreover, the number of autoantibody reactivities correlated with neither double-negative B cell populations nor days from acute symptom onset (Extended Data Fig. 7a,b).

Given REAP studies showing that specific functional autoantibodies are elevated in severe acute COVID-19^16^, autoantibody reactivities were aggregated into clusters using a manually curated Gene Ontology process list relevant to LC. The magnitudes of reactivity for LC and control groups did not significantly differ in any category (Extended Data Fig. 7c). Several reports implicated stereotypical G-protein-coupled receptor (GPCR) autoantibodies in LC pathogenesis^31,32^ (for example, targeting β-adrenergic receptors or the angiotensin II receptor). While several GPCR-directed autoantibodies were detected in this study (Extended Data Fig. 7d), the number of GPCR reactivities for participants with LC did not differ from that of the controls (Fig. 3d). Importantly, there were no individual autoantibody reactivities that were significantly more frequent in either participants with LC or in controls (Fig. 3e).

## Antibody responses to herpesviruses

**Fig. 4:**
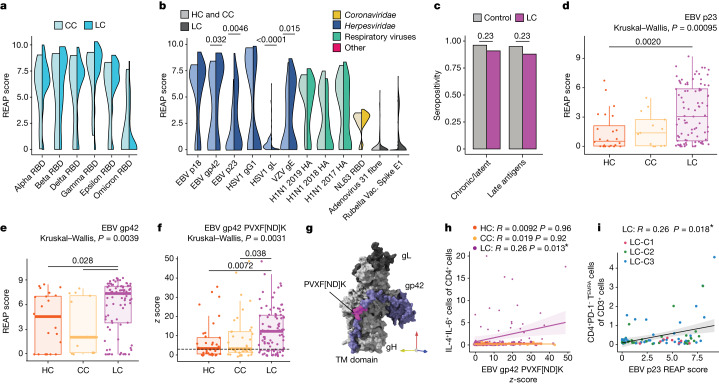
Participants with LC demonstrate elevated levels of antibody responses to herpesviruses. **a**, The REAP score distributions for SARS-CoV-2 S1 RBD between participants in the LC (*n* = 69) and CC (*n* = 10) groups with two doses of mRNA vaccine. Statistical significance was assessed using Wilcoxon rank-sum tests adjusted for multiple comparisons using the Benjamini–Hochberg method. **b**, The REAP score distributions for a given viral antigen between participants in the LC (*n* = 98) and pooled control (HC and CC, *n* = 38) groups. Statistical significance was assessed using Wilcoxon rank-sum tests adjusted for multiple comparisons using the Benjamini–Hochberg method. Only antigens with ≥2 individuals with LC and ≥2 control individuals with REAP score ≥ 1 were included. **c**, Seropositivity as assessed by SERA for EBV among participants with LC (*n* = 99) and control participants (*n* = 78). Significance was assessed using Fisher’s exact tests adjusted for multiple comparisons using the Benjamini–Hochberg method. **d**,**e**, REAP scores among EBV-seropositive individuals only for EBV p23 (**d**) and gp42 (**e**) by group. *n* = 25 (HC), *n* = 13 (CC), *n* = 98 (LC). **f**, SERA-derived *z* scores for the gp42 motif PVXF[ND]K among EBV-seropositive individuals only, plotted by group. The dashed line represents the *z*-score threshold for epitope positivity defined by SERA. *n* = 39 (HC), *n* = 38 (CC) and *n* = 80 (LC). **g**, Three-dimensional mapping of the LC-enriched linear peptide sequence PVXF[ND]K (magenta) onto EBV gp42 (purple) in a complex with gH (light grey) and gL (dark grey) (PDB: 5T1D). **h**, The relationship between the EBV gp42 PVXF[ND]K *z* score and the percentage of IL-4/IL-6 double-positive CD4^+^ T cells (of total CD4^+^ T cells) for participants. Only EBV-seropositive individuals were included. Correlation was assessed using Spearman’s correlation. The black line shows linear regression, and the shading shows the 95% CIs. *n* = 39 (HC), *n* = 38 (CC) and *n* = 80 (LC). **i**, The relationship between EBV p23 REAP score and the percentage of CD4^+^ T_EMRA_ cells (of total CD3^+^ T cells). Only EBV-seropositive individuals were included. Correlation was assessed using Spearman’s correlation. The black line depicts linear regression, and the shading shows the 95% CIs. Colours depict LCPS clusters as in Fig. 3. For the box plots, the central lines indicate the group median values, the top and bottom lines indicate the 75th and 25th percentiles, respectively, the whiskers represent 1.5× the interquartile range. Each dot represents one individual. Statistical significance of the difference in median values was determined using Kruskal–Wallis tests. Post hoc tests were performed using Dunn’s test with Bonferroni–Holm’s method to adjust for multiple comparisons. TM, transmembrane.

**Extended Data Fig. 8:**
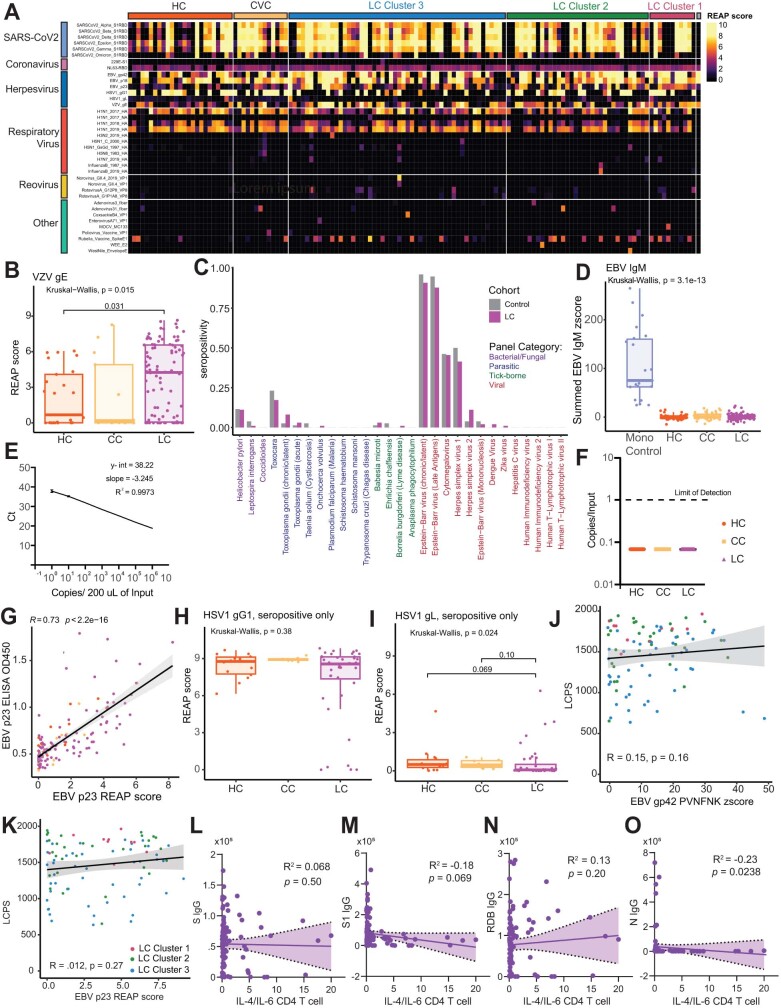
Non-SARS-CoV-2 humoral responses among participants with Long COVID. (**A**) Heatmap depicting REAP reactivities to viral antigens across the MY-LC cohort. Each column is one participant, grouped by control or LCPS cluster. Column clustering within groups performed by K-means clustering. Each row is one viral protein. Reactivities depicted have at least one participant with a REAP score >= 2. (**B**) REAP scores for VZV gE by group (HC = healthy control; CC = convalescent control; LC = Long COVID). Statistical significance determined by Kruskal Wallis with correction for multiple comparison using Bonferroni-Holm. Each dot represents one individual (n = 25 HC, n = 13 CC, n = 98 LC). Bottom and top lines depict 25^th^ to 75^th^ percentile of the data, with the middle line representing the median. Whiskers represent 1.5x the inter-quartile range (IQR). (**C**) Proportion of each group (LC: n = 99, control: n = 78) seropositive for each of 30 common pathogen panels as determined by SERA, grouped by pathogen-type (LC = Long COVID). Statistical significance determined by Fisher’s exact test corrected with FDR (Benjamini Hochberg). (**D**) Sum of SERA-derived z-scores for IgM reactivity to EBV antigens plotted by group. Statistical significance determined by Kruskal-Wallis with correction for multiple comparison using Bonferroni-Holm. Each dot represents one individual (n = 22 Mono-control, n = 40 HC, n = 38 CC, n = 98 LC). Boxplot coloured box depicts 25^th^ to 75^th^ percentile of the data, with the middle line representing the median. Whiskers represent 1.5× the inter-quartile range (IQR). (**E**) Standard curve for Taqman PCR of EBV *BNRF1*. Serial dilutions of EBV standard ranging from 1 to 10^6^ copies per 200 μL input material were made. C_t_ values are plotted against standard copy number, demonstrating ability to detect 1 genome copy. (**F**) Copies of EBV genome detected in participant serum by Taqman PCR for EBV *BNRF1* plotted by group. All samples were below the limit of detection. (**G**) Correlation plot depicting the relationship between EBV p23 REAP score and EBV p23 ELISA O.D. 450 nm. Correlation assessed by Spearman. Black line depicts linear regression with 95% CI shaded. Colours depict group (purple, LC; yellow, CC; orange, HC). Each dot represents one individual. (**H**,**I**) REAP scores for HSV1 gD1 (H) and HSV1 gL (I) amongst HSV1 seropositive individuals only, separated by group (HC = healthy control; CC = convalescent control; LC = Long COVID). Statistical significance determined by Kruskal Wallis with correction for multiple comparison using Bonferroni-Holm. Each dot represents one individual. Boxplot coloured box depicts 25^th^ to 75^th^ percentile of the data, with the middle line representing the median. Whiskers represent 1.5× the inter-quartile range (IQR). Each dot represents one individual. (**J**,**K**) Correlation plot depicting the relationship between Long COVID Propensity Score (LCPS) and EBV gp42 PVXF[ND]K (J) or EBV p23 REAP score (K). Correlation assessed by Spearman. Each dot represents one individual. Colours depict Long COVID cluster (cluster 1, blue; cluster 2, green; cluster 3, red). Black line depicts the linear regression, with the 95% CI shaded. (**L**-**O**) Linear regressions of various SARS-CoV-2 antigens and IL-4/IL-6 double positive CD4 T cells. Spearman’s correlation were calculated for each pair of variables, with corresponding p-values reported. Black lines depict linear regressions with the shaded area representing the 95% CI.

Given emerging evidence for the role of latent virus reactivation in LC, three complementary approaches were used to examine anti-viral reactivity patterns in the MY-LC cohorts: REAP, serum epitope repertoire analysis (SERA) and enzyme-linked immunosorbent assay (ELISA). Global anti-viral responses were first assessed using REAP, which measures antibody reactivity to 225 viral surface proteins (Supplementary Table 2). Reactivities against 38 viral conformational epitopes were detected among 98 LC and 38 control participants (Extended Data Fig. 8a). For SARS-CoV-2 reactivities, only participants who received two doses of vaccine were analysed. Reactivities against non-Omicron-variant RBDs in the LC cohort were higher than those in the CC controls (Fig. 4a), however this trend was not significant.

Differences in viral reactivities against non-SARS-CoV-2 antigens were marked (Fig. 4b). Participants with LC had elevated REAP scores for several herpesvirus antigens, including the Epstein–Barr virus (EBV) minor viral capsid antigen gp23 (*P* = 4.62 × 10^−3^), the EBV fusion-receptor component gp42 (*P* = 3.2 × 10^−2^) and the varicella zoster virus (VZV) glycoprotein E (*P* = 1.51 × 10^−2^) (Extended Data Fig. 8b). Conversely, participants with LC had lower REAP scores for HSV-1 glycoprotein gL (*P* = 4.61 × 10^−6^) and gD1, although the difference in gD1 reactivity was not significant.

Next, the SERA platform (a commercially available random bacterial display library with unlimited multiplex capability) was used to orthogonally analyse non-SARS-CoV-2 antigens. SERA includes epitope panels representing 45 pathogens and disease markers, validated using a database of thousands of controls^33^. Importantly, SERA revealed that cohorts significantly differed neither in estimated EBV seroprevalence (Fig. 4c) nor for any other tested viral pathogen (Extended Data Fig. 8c).

First, we assessed whether individuals with LC had higher EBV reactivities because of acute EBV infection. Anti-EBV IgM was not elevated in this group (as measured by SERA) (Extended Data Fig. 8d) nor was there evidence of EBV viraemia (Extended Data Fig. 8e,f), suggesting that the higher reactivity to EBV lytic antigens was more probably caused by recent EBV reactivation than by acute infection. Furthermore, these results do not rule out EBV shedding at a local site, such as in the saliva^34^.

We next assessed whether differences in baseline seropositivity affected EBV-antigen reactivity. EBV reactivity was analysed only in EBV-seropositive individuals as identified by SERA and using identifying motifs using next-generation sequencing (NGS) experiments (IMUNE). On the basis of REAP, seropositive participants with LC had significantly higher reactivity to EBV p23 (Kruskal–Wallis, *P* = 0.00095; Fig. 4d) and gp42 (0.0039; Fig. 4e) compared with the seropositive controls. REAP measurements significantly correlated with ELISA measurements (*R* = 0.73, *P* ≤ 2.2 × 10^−16^), orthogonally validating this finding (Extended Data Fig. 8g). In an orthogonal screen of linear peptides with SERA, the LC cohort had greater reactivity against the gp42 linear peptide (PVXF[ND]K) (Kruskal–Wallis, *P* = 0.0031) (Fig. 4f). Mapping of this motif onto available structures of gp42 complexed with EBV gH/gL (PDB: 5T1D) showed that these residues are exposed on the surface of EBV virions (Fig. 4g (pink residues)).

To investigate lower REAP reactivity to HSV-1 antigens observed in participants with LC, a similar analysis was performed using only HSV-1-seropositive individuals, as identified by SERA. In these individuals, REAP scores for HSV-1 glycoprotein gD1 no longer differed among groups (Extended Data Fig. 8h). Post hoc comparisons for HSV-1 gL also showed that the groups did not significantly differ (Extended Data Fig. 8i). These data suggest that the lower IgG reactivity to gL in REAP (Fig. 4b) is probably caused by lower HSV-1 seroprevalence in the LC group. In aggregated initial REAP and SERA results, individuals with LC had elevated IgG reactivity to EBV and VZV surface antigens without evidence of EBV primary infection or acute viraemia.

Additional analysis showed no correlation between LCPS and humoral reactivity against gp42 PVXF[ND]K or EBV p23 antigens in EBV-seropositive individuals (Extended Data Fig. 8j,k). By contrast, reactivity to gp42 PVXF[ND]K correlated with IL-4/IL-6 producing CD4^+^ T cells in EBV-seropositive individuals with LC (*R* = 0.26, *P* = 0.013) (Fig. 4h). This correlation was not observed in the control groups. Furthermore, EBV p23 REAP reactivity significantly correlated with terminally differentiated effector memory (T_EMRA_) CD4^+^ T cells (*R* = 0.26, *P* = 0.018) (Fig. 4i), a subset of cells implicated in protection from cytomegalovirus^35^. By contrast, anti-SARS-CoV-2 antibody levels did not correlate with IL-4/IL-6 double-positive CD4^+^ T cells (Extended Data Fig. 8l–o).

## Unique biological markers of LC

**Fig. 5:**
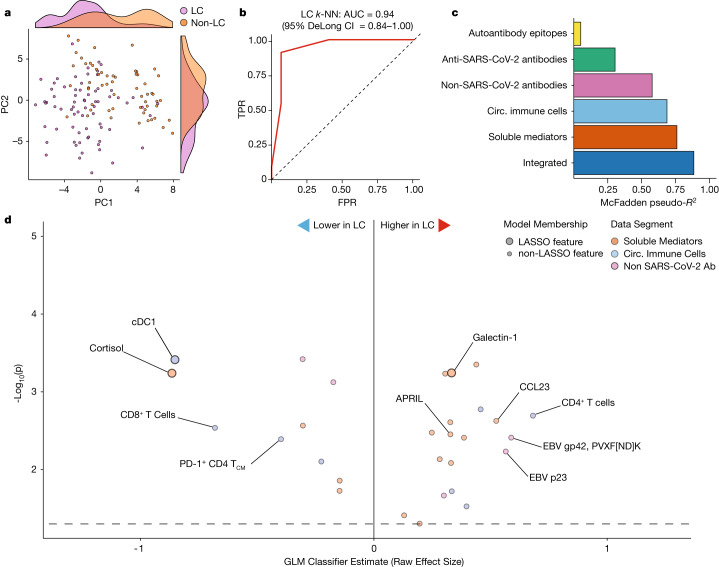
Biochemical factors differentiate participants with LC from the matched controls. All data shown represent a matched subset of participants (*n* = 40 (HC), *n* = 39 (CC) and *n* = 79 (LC)) selected using the Gale–Shapley procedure on demographic factors (Extended Data Fig. 9a). **a**, PCA projection of participant data comprising cytokine, flow cytometry and various antibody responses (anti-SARS-CoV-2, non-SARS-CoV-2 viral antibodies and autoantibodies (aAb)). Marginal histograms display data density along each principal component dimension. **b**, Receiver operating characteristic curve analysis from unsupervised *k*-NN classification. AUC and 95% CI intervals (DeLong’s method) are reported. **c**, McFadden’s pseudo-*R*^2^ values are reported as a bar plot for each data segment. An integrated, parsimonious McFadden’s pseudo-*R*^2^ is reported for the final classification model (all). **d**, LASSO regression identifies a minimal set of immunological features differentiating participants with LC from others. Unlabelled dots are significant predictive features that were not included in the final LASSO regression model. Dots are coloured according to individual data segments: orange, flow; blue, plasma cytokines; pink, viral epitopes; green, anti-SARS-CoV-2; yellow, autoantibodies. Flow, flow cytometry; FPR, false-positive rate; T_CM_, T central memory cells; TPR, true-positive rate.

**Extended Data Fig. 9:**
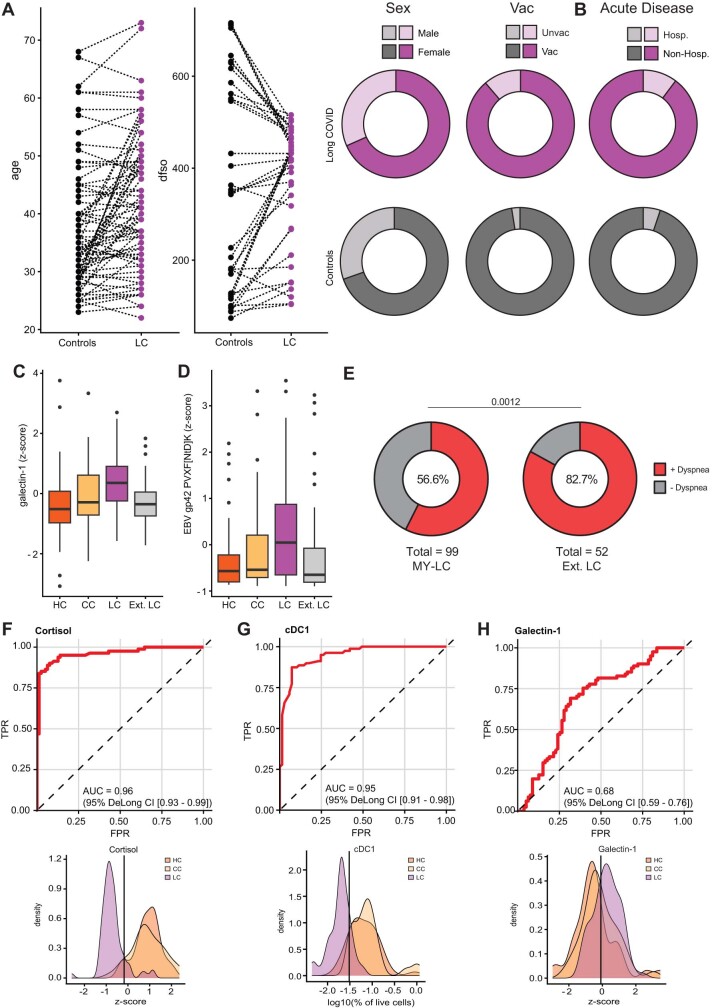
Gale-Shapley matching of Long COVID group and controls harmonizes samples by disease and demographics characteristics. (**A**) Features used in the preference list construction for Gale-Shapley matching are shown. Individual paired samples are shown for participant age and days from initial acute COVID-19 infection (dfso). Paired plots for sex and vaccination status are shown. (**B**) Additionally, differences between populations in the severity of initial acute COVID-19 infection are shown. No differences between groups are significant by a Chi-square test. (**C**,**D**) Box plots of selected features assessed in the Ext. LC group. Centre lines represent median values with error bars representing 1.5 standard deviation. (**E**) Distribution of respiratory symptoms (“dyspnea” or “shortness of breath”) between individuals with Long COVID in the MY-LC study and the Ext. LC group. Significance was assessed using Fisher’s exact test. (**F**–**H**) ROC curve analysis using cortisol, cDC1, and galectin-1 levels as an individual classifier of Long COVID status. AUC and 95% CI intervals (DeLong’s Method) for each feature are displayed (top). Kernel-density smoothed histograms for HC, CC and LC cohorts for selected model predictors. Vertical lines depict threshold values for each feature with maximal discriminatory accuracy (bottom).

To further account for demographic differences among groups that might affect immunophenotypes, each participant with LC was explicitly matched to a control participant using a Gale–Shapley procedure based on participant age, sex, days from acute COVID-19 symptom onset and vaccination status. Participants with LC did not differ significantly from controls in these criteria (Extended Data Fig. 9a), nor in the severity of acute COVID-19 disease (whether hospitalization was required) (Extended Data Fig. 9b). Principal component analysis (PCA) embedding of matched participants with all collected immunological features clearly distinguished individuals with LC from the controls (Fig. 5a). Consistent with this, *k*-nearest neighbour (*k*-NN) classification of the normalized features efficiently discriminated between groups, with an AUC of 0.94 (95% CI = 0.84–1.00) (Fig. 5b). Principal component regression of collated immunological data showed that flow cytometry (pseudo-*R*^2^ = 59%) and plasma proteomics and hormones (pseudo-*R*^2^ = 74%) were the most informative for separating groups. A final parsimonious LASSO model similarly achieved a good fit (pseudo-*R*^2^ = 82%) (Fig. 5c). Of the features selected for the final model, several associated positively with LC status (serum galectin-1 concentration, IgG against various EBV epitopes), while others associated negatively (serum cortisol, PD-1^+^CD4^+^ T central memory cells, cDC1 cells) (Fig. 5d). Preliminary external validation in the EXT-LC cohort of selected LASSO-model features revealed similar decreases in cortisol, but galectin-1 and EBV gp42 predicted LC status specifically in the MY-LC cohort (Extended Data Fig. 9c,b), potentially caused by clinical phenotype differences between the MY-LC and EXT-LC cohorts (Extended Data Fig. 9e).

Serum cortisol was the most significant predictor of LC status in the model, and cortisol alone achieved an AUC of 0.96 (95% CI = 0.93–0.99) (Extended Data Fig. 9f (top)). Notably, serum cortisol in the MY-LC cohort was similar in the HC and CC control groups, and lower in participants with LC (Extended Data Fig. 9f (bottom)). When used alone, each of the other selected model features predicted status reasonably well (Extended Data Fig. 9g,h). Finally, classification accuracies of LCPS models, determined using the maximum Youden’s *J* index, largely agreed with machine learning ones (Cohen’s *κ* = 0.52; 95% CI = 0.33–0.72), suggesting that both participant-reported outcomes and immunological features efficiently predict LC status (Extended Data Table 7).

## Discussion

Studies of individuals with LC reported diverse changes in immune and inflammatory factors^36,37^. In this study, exploratory analyses identified significant immunological differences between individuals with LC and demographically matched control populations more than a year after their acute infections. Circulating immune cell populations were significantly changed. Populations of non-conventional monocytes, double-negative B cells and IL-4/IL-6-secreting CD4^+^ T cells increased, and those of conventional DC1 and central memory CD4^+^ T cells decreased. Moreover, individuals with LC had higher levels of antibodies to SARS-CoV-2, EBV and VZV antigens. By contrast, the levels of individual autoantibodies to human exoproteome did not significantly differ. Marked differences in the levels of circulating cytokines and hormones, particularly cortisol, were noted in participants with LC from both the MY-LC and EXT-LC cohorts. Unbiased machine learning revealed several core predictive features of LC status within the MY-LC study, identifying potential targets for additional validation and future biomarker development.

Multiple hypotheses have been proposed for LC pathogenesis, including persistent virus or viral remnants^38^, autoimmunity, dysbiosis, latent viral reactivation and unrepaired tissue damage. The data in this study suggest that persistent SARS-CoV-2 viral antigens, reactivation of latent herpesviruses and chronic inflammation may all contribute to LC. Overall, our data are less consistent with an autoantibody-dominated disease process in LC. Whether autoreactive T cells have a role in LC pathogenesis was not addressed and requires future investigation.

Immune phenotyping of PBMC populations revealed that participants with LC had notably higher levels of circulating non-conventional monocytes associated with various chronic inflammatory and autoimmune conditions^39^. These participants also had significantly lower levels of circulating cDC1 populations, which are responsible for antigen presentation and cytotoxic T cell priming^40^. Moreover, the number of CD4^+^ T central memory cells was significantly reduced and the absolute number of exhausted CD4^+^ T cells was increased. Cerebrospinal fluid from individuals with LC also has elevated levels of TIGIT^+^CD8^+^ T cells, consistent with possible immune exhaustion^41^. After stimulation, T cells from individuals with LC produced significantly more intracellular IL-2 (CD4^+^ and CD8^+^ T cells), IL-4 (CD4^+^ T cells) and IL-6 (CD8^+^ T cells). Notably, subsets of participants with LC also had polyfunctional IL-4/IL-6-co-expressing CD4^+^ T cells, which correlated with reactivity against EBV lytic antigens, but not against SARS-CoV-2 antigens. Together, these findings may be consistent with T-helper-2-cell-skewed CD4^+^ T cell activation in response to EBV among participants with LC, as suggested for myalgic encephalomyelitis/chronic fatigue syndrome^42^. The levels of IgG against SARS-CoV-2 spike and S1 in participants with LC were also higher compared with those in vaccination-matched controls, consistent with persistent viral antigens^43–45^.

Participants with LC from two sites had significantly decreased systemic cortisol levels; this remained significant after accounting for variations in demographics and sample-collection times. Notably, the decreased cortisol did not associate with a compensatory increase in ACTH levels, suggesting that the hypothalamic–pituitary axis response to regulate cortisol may be inappropriately blunted. Importantly, ACTH has an extremely short half-life in the plasma, which may impair accurately detecting changes. Dedicated studies must confirm these preliminary findings. Notably, an earlier study of 61 individuals who survived SARS-CoV infection showed similar evidence of hypocortisolaemia and blunted ACTH responses 3 months after acute disease^46^. Furthermore, decreased cortisol levels during the early phases of COVID-19 were associated with development of respiratory LC symptoms^47^. As cortisol is central for a variety of homeostatic and stress responses^48^, the current finding of persistently lower cortisol levels in those with LC more than a year after acute infection warrants further investigations.

We also showed that individuals with LC have elevated antibody responses against non-SARS-CoV-2 viral antigens, particularly EBV antigens. EBV viraemia occurs during acute COVID-19 in hospitalized patients and predicts development of persistent symptoms in the post-acute period^47^. Other studies implicated recent EBV reactivation with LC development^49,50^. The observation here of elevated IgG against EBV lytic antigens suggests that recent reactivation of latent herpesviruses (EBV and VZV) may be a common feature of LC.

Finally, machine learning models designed to accurately classify LC and control populations (after matching individuals to account for potentially confounding features, such as sex, age, days from symptom onset and vaccination status) identified multiple features that significantly predict LC status. Classifications using only immunological data agreed with classifications using survey scores, showing that the immunological analyses and patient-reported outcomes used here were concordant in diagnosing LC.

This study has several limitations. Primary among these is that few participants were identified by convenience sampling and that recruitment strategies for cases differed from those for controls. Although broadly covering diverse biological features, this study used far fewer independent observations than traditional machine learning studies use (several thousands) to robustly train and optimize classification models. This study was also restricted to analysing peripheral (circulating) immune factors from participants. As LC often presents with organ-system-specific dysfunctions, greater analyses of local immune features would crucially extend these findings. Furthermore, analysis of autoantibodies was restricted to the exoproteome. Whether autoantibodies to intracellular antigens or non-protein antigens participate in LC pathogenesis was not tested.

In summary, significant biological differences were identified between participants with LC and demographically and medically matched CC and HC participants, validating extensive reports of persistent symptoms by various individuals with LC and patient advocacy groups. This study provides a basis for future investigations into the immunological underpinnings driving the genesis of LC.

## Methods

### Ethics statement

This study was approved by the Mount Sinai Program for the Protection of Human Subjects (IRB 20-01758) and Yale Institutional Review Board (IRB 2000029451 for MY-LC; IRB 2000028924 for enrolment of pre-vaccinated Healthy Controls; HIC 2000026109 for EXT-LC). Informed consent was obtained from all enrolled participants.

### MY-LC study design, enrolment strategy and inclusion/exclusion criteria

MY-LC was a cross-sectional, multi-site study comprising five different groups with differing SARS-COV-2 exposure histories and varied LC status. The participants who enrolled in the LC group underwent complete medical evaluations by physicians to rule out alternative medical aetiologies for their persistent symptoms before study enrolment.

Participants with persistent symptoms following acute COVID-19 were recruited from LC clinics within the Mount Sinai Healthcare System and the Centre for Post COVID Care at Mount Sinai Hospital. Participants enrolled in healthy and convalescent study arms were recruited through IRB-approved advertisements delivered through email lists, study flyers located in hospital public spaces, and on social media platforms. Informed consent was provided by all of the participants at the time of enrolment. All of the participants provided peripheral blood samples and completed symptom surveys on the day of sample collection (described below). Self-reported medical histories for all of the MY-LC cohort participants were also collected at study visits and further reviewed through examination of electronic medical records by collaborating clinicians.

Inclusion criteria for individuals in the LC group were age ≥ 18 years; previous confirmed or probable COVID-19 infection (according to World Health Organization guidelines^51^); and persistent symptoms >6 weeks after initial COVID-19 infection. Inclusion criteria for enrolment of individuals in the HC group were age ≥ 18 years, no previous SARS-CoV-2 infection, and completion of a brief, semi-structured verbal screening with research staff confirming no active symptomatology. Inclusion criteria for individuals in the CC group were age ≥ 18 years; previous confirmed or probable previous COVID-19 infection; and completion of a brief, semi-structured verbal screening with research staff confirming no active symptomatology.

Pre-specified exclusion criteria for this study were inability to provide informed consent; and any condition preventing a blood test from being performed. Furthermore, all of the participants had their electronic health records reviewed by study clinicians after enrolment and were subsequently excluded before the analyses for the following reasons: (1) current pregnancy; (2) immunosuppression equivalent to or exceeding prednisone 5 mg daily; (3) active malignancy or chemotherapy; and (4) any monogenic disorders. For specific immunological analyses, pre-existing medical conditions were also excluded before analyses due to high potential for confounding (for example, participants with hypothyroidism were excluded before analysis of circulating T3/T4 levels; and participants with pituitary adenomas were excluded before analysis of cortisol levels). Specific exclusions are marked by a triangle in the figures and detailed in the relevant legends.

The recruitment of individuals in the HCW group was described at length previously^52^. Individuals in the EXT-LC cohort were identified from The Winchester Centre for Lung Disease’s Post-COVID-19 Recovery Program at Yale New Haven Hospital by collaborating clinicians. All of the participants underwent medical evaluation for persistent symptoms after COVID-19. Participants from this group were identified retrospectively for inclusion in the MY-LC study according to the established MY-LC protocol: age ≥ 18 years; previous confirmed or probable COVID-19 infection (according to World Health Organization guidelines^39^); and persistent symptoms >6 weeks after initial COVID-19 infection.

### Participant surveys

A comprehensive suite of surveys was administered to MY-LC study participants, combining validated patient-reported outcomes with custom, purpose-developed tools by the MY-LC study team. Baseline demographic data collected from surveys included gender, age, BMI, race and medical comorbidities. Furthermore, participants in the LC and CC groups were asked to provide COVID-19 clinical data including date of symptom onset and acute disease severity (non-hospitalized versus hospitalized), any SARS-CoV-2 PCR diagnostic testing results and any SARS-CoV-2 antibody testing results. Finally, all of the participants were asked to report SARS-CoV-2 vaccination status, including the date of vaccinations and vaccine brand.

At the time of blood collection, all of the participants completed patient-reported outcomes for fatigue (fatigue severity scale (FSS))^53^, fatigue visual analogue scale), post-exertional malaise (DePaul symptom questionnaire post-exertional malaise short form (DSQ-PEM short form))^54^, breathlessness (Medical Research Council (MRC) breathlessness scale^55^), cognitive function (Neuro-QOL v.2.0 cognitive function short form^56^), health-related quality of life (EuroQol EQ-5D-5L^57^), anxiety (GAD-7)^58^, depression (PHQ-2)^59^, pain visual analogue scale, sleep (single-item sleep quality scale^60^), as well as pre- and post-COVID-19 employment status (author developed). Finally, the participants in the MY-LC study were asked to self-report any current persistent symptoms from a study-provided list.

All survey data were collected and securely stored using REDCap^61,62^ (Research Electronic Data Capture) electronic data capture tools hosted within the Mount Sinai Health System.

### LCPS

Calculation of propensity scores for each participant was achieved through construction of a multivariable logistic regression model generated with LC versus others (HC + CC) as the outcome. The model candidate variables included survey responses from the following instruments described previously: FSS, fatigue visual analogue scale, DSQ-PEM short form, MRC breathlessness scale, Neuro-QOL v2.0 cognitive function short form, EQ-5D-5L, GAD-7, PHQ-2, pain visual analogue scale and single-item sleep quality scale. Model selection using Akaike’s information criteria was used to select the final, parsimonious model. Odds ratios from the final model were normalized by dividing them by their respective standard error (s.e.) and rounding off to the nearest integer. These integer values were considered to be the score items for these specific variables and a cumulative prediction score for each participant was calculated by summation (equation (1)). As the score did not significantly differ between HC and CC individuals, the two control groups were combined as a single group (others) for final analysis. A ROC curve analysis was performed to identify the optimal cut-off for the LCPS using the maximum value of Youden’s index *J* for LC versus others. A tenfold cross-validation was used for internal validation and to obtain 95% CIs for the AUC. Data were analysed using Stata v.16 (StataCorp).

1 $${\rm{LCPS}}=7\times \sum {\rm{GAD}}+1\times \sum {\rm{MRC}}+2\times \sum {\rm{PHQ}}2+3\sum {\rm{EQ}}5+28\times \sum {\rm{FSS}}.$$

### Blood sample processing

Whole blood was collected in sodium-heparin-coated vacutainers (BD 367874, BD Biosciences) from participants at Mount Sinai Hospital in New York City, New York. After blood draw, all of the participant samples were assigned unique MY-LC study identifiers and de-identified by clinical staff. The samples were couriered directly to Yale University in New Haven, CT, on the same day as the sample collection. Blood samples were processed on the same day as collection. Plasma samples were collected after centrifugation of whole blood at 600*g* for 10 min at room temperature without braking. Plasma was then transferred to 15 ml polypropylene conical tubes, aliquoted and stored at −80 °C. The PBMC layer was isolated according to the manufacturer’s instructions using SepMate tubes (StemCell). Cells were washed twice with phosphate-buffered saline (PBS) before counting. Pelleted cells were briefly treated with ACK lysis buffer (Thermo Fisher Scientific) for 2 min and then counted. Viability was estimated using standard Trypan blue staining and a Countess II automated cell counter (Thermo Fisher Scientific). PBMCs were stored at −80 °C for cryopreservation or plated directly for flow cytometry studies. Plasma samples from the EXT-LC group were obtained using BD Vacutainer CPT tubes (362753) according to the manufacturer’s instructions and stored in aliquots at −80 °C before analysis.

### Flow cytometry

Freshly isolated PBMCs were plated at 1–2 × 10^6^ cells per well in a 96-well U-bottom plate. Cells were resuspended in Live/Dead Fixable Aqua (Thermo Fisher Scientific) for 20 min at 4 °C. Cells were washed with PBS and followed by Human TruStain FcX (BioLegend) incubation for 10 min at room temperature. Cocktails of staining antibodies were added directly to this mixture for 30 min at room temperature. Before analysis, cells were washed and resuspended in 100 μl 4% PFA for 30 min at 4 °C. For intracellular cytokine staining after stimulation, the surface-marker-stained cells were resuspended in 200 μl cRPMI (RPMI-1640 supplemented with 10% FBS, 2 mM l-glutamine, 100 U ml^−1^ penicillin, and 100 mg ml^−1^ streptomycin, 1 mM sodium pyruvate) and stored at 4 °C overnight. Subsequently, these cells were washed and stimulated with 1× cell stimulation cocktail (eBioscience) in 200 μl cRPMI for 1 h at 37 °C. A total of 50 μl of 5× stimulation cocktail in cRPMI (plus protein transport 442 inhibitor, eBioscience) was added for an additional 4 h of incubation at 37 °C. After stimulation, cells were washed and resuspended in 100 μl 4% paraformaldehyde for 30 min at 4 °C. To quantify intracellular cytokines, cells were permeabilized with 1× permeabilization buffer from the FOXP3/Transcription Factor Staining Buffer Set (eBioscience) for 10 min at 4 °C. All of the subsequent staining cocktails were made in this buffer. Permeabilized cells were then washed and resuspended in a cocktail containing Human TruStain FcX (BioLegend) for 10 min at 4 °C. Finally, intracellular staining cocktails were added directly to each sample for 1 h at 4 °C. After this incubation, cells were washed and prepared for analysis on the Attune NXT (Thermo Fisher Scientific) system. Data were analysed using FlowJo v.10.8 (BD). Antibody information is provided in Supplementary Table 1.

A PERMANOVA test was used to assess the relationship between all circulating immune cell populations presented in Extended Data Fig. 2 and participant age, sex, LC status and BMI. The PERMANOVA test was run using the vegan package in R^63^.

### SARS-CoV-2 antibody testing using ELISA

ELISAs were performed as previously described^15^. In brief, Triton X-100 and RNase A were added to plasma samples at final concentrations of 0.5% and 0.5 mg ml^−1^, respectively, and incubated at room temperature for 30 min before use to reduce the risk from any potential virus in the plasma. MaxiSorp plates (96 wells; 442404, Thermo Fisher Scientific) were coated with 50 μl per well of recombinant SARS-CoV-2 Total S (SPN-C52H9 100 μg, ACROBiosystems), RBD (SPD-C52H3 100 μg, ACROBiosystems) and the nucleocapsid protein (NUN-C5227 100 μg, ACROBiosystems) at a concentration of 2 μg ml^−1^ in PBS and were incubated overnight at 4 °C. The coating was removed, and the plates were incubated for 1 h at room temperature with 200 μl of blocking solution (PBS with 0.1% Tween-20 and 3% milk powder). Plasma was diluted serially at 1:100, 1:200, 1:400 and 1:800 in dilution solution (PBS with 0.1% Tween-20 and 1% milk powder), and 100 μl of diluted serum was added for 2 h at room temperature. Human anti-spike (SARS-CoV-2 human anti-spike (AM006415, 91351, Active Motif) and anti-nucleocapsid SARS-CoV-2 human anti-nucleocapsid (1A6, MA5-35941, Active Motif) were serially diluted to generate a standard curve. The plates were washed three times with PBS-Tween (PBS with 0.1% Tween-20) and 50 μl of HRP anti-human IgG antibody (1:5,000; A00166, GenScript) added to each well in dilution solution. After 1 h of incubation at room temperature, the plates were washed six times with PBS-Tween. The plates were developed with 100 μl of the TMB Substrate Reagent Set (555214, BD Biosciences) and the reaction was stopped after 5 min by the addition of 2 N sulfuric acid. Plates were then read at an excitation/emission wavelength of 450 nm and 570 nm, respectively.

### Multiplex proteomic analysis

Participant plasma was isolated and stored at −80 °C as described above. Plasma was shipped to Eve Technologies on dry ice and analytes were measured using the following panels: Human Cytokine/Chemokine 71-plex Discovery Assay (HD71), Steroid/Thyroid 6plex Discovery Assay (STTHD), TGF-Beta 3-plex Discovery Assay (TGFβ1-3), Human Myokine Assay (HMYOMAG-10), Human Neuropeptide Assay (HNPMAG-05), Human Pituitary Assay (HPTP1), Human Cytokine P3 Assay (HCYP3-07), Human Cytokine Panel 4 Assay (HCYP4-19), Human Adipokine Panel 2 Assay (HADK2-03), Human Cardiovascular Disease Panel Assay (HDCVD9), Human CVD2 Assay (HCVD2-8), Human Complement Panel Assay (HDCMP1) and Human Adipokine Assay (HDADK5). Analysis of plasma proteomics was completed in two batches with internal controls in each shipment to assess for and correct any analyte batch effects (described below).

To integrate analytes across batches, two samples from the same representative individuals from each group (2 from LC, 2 from CC and 2 from HC) were measured in each analysis batch. The median difference between all paired samples between the first and second batch was used as an additive corrective factor to integrate samples across batches. After batch integration, each feature was *z*-scored using all measurements across both batches. After *z*-scoring, features that were found to have persistent batch effects, as defined by a Wilcoxon rank-sum test *P* < 0.05 after correction, were not considered for downstream analysis.

### Real-time TaqMan assay for the detection of EBV DNA

#### Nucleic acid extraction

Nucleic acid was extracted from 200 μl freeze–thawed serum using the MagMAX Viral/Pathogen Nucleic Acid Isolation Kit (Thermo Fisher Scientific, A42352), automated on the KingFisher Flex (Thermo Fisher Scientific) system according to the manufacturer’s protocol. The manufacturer’s protocol was additionally modified to reduce salt carry-over by adding a third wash step with 500 μl 80% ethanol and eluting in 50 μl nuclease-free water.

#### Real-time TaqMan PCR

PCR primers for the TaqMan assay were previously validated^64^: EBV p143 forward (5′-GGAACCTGGTCATCCTTGC) and EBV p143 reverse (5′-ACGTGCATGGACCGGTTAAT) (Thermo Fisher Scientific). A fluorogenic probe (5′-(FAM)-CGCAGGCACTCGTACTGCTCGCT-(MGB)-3′) with a FAM reporter molecule attached to the 5′ end and an MGB quencher linked at the 3′ end was acquired in parallel (Thermo Fisher Scientific). The PCR amplification was performed in a 20 μl volume containing 10 μl 2× Luna Universal Probe One-Step RT-qPCR Kit (New England BioLabs), 300 pmol of each primer per μl, 200 pmol of the TaqMan probe and 5 μl of isolated DNA. Amplification and detection were performed on the CFX96 Touch instrument (Bio-Rad). After a 1 min hold step at 95 °C, the PCR cycling program consisted of 42 two-step cycles of 15 s at 95 °C and 30 s at 60 °C. Real-time measurements were taken, and a threshold cycle (*C*_t_) value for each sample was calculated if the fluorescence exceeded a threshold limit. Each sample was run in duplicate and was considered to be positive only if both replications were above the threshold limit. Each run contained multiple H_2_O controls (no template), and a standard curve containing serial dilutions of quantitative synthetic DNA (described below, ATCC, VR-3247SD). An additional EBV plasma control was included as a positive control for each assay plate (Thermo Fisher Scientific, 961231).

#### Estimating genome copy-number standards

For standardization of quantitative PCR (qPCR) detection of EBV viral genomes from participant plasma, a commercially available standard containing 5.59 × 10^8^ EBV genome copies per ml (ATCC, VR-3247SD) was used. Serial log dilutions of this standard, ranging from 10^6^ to 10^0^ copies per ml, were made to establish the sensitivity of the TaqMan qPCR and included on each assay plate. The standard curve was created in the usual way by plotting the *C*_t_ values against the standard of known concentration. *x–y* scatter diagrams were drawn, and the correlation coefficient (*r*^2^) was determined. Linear regression analysis was performed using GraphPad Prism.

### Linear peptide profiling

#### SERA serum screening

A detailed description of the SERA assay has been published previously^33^. For this study, plasma was incubated with a fully random 12-mer bacterial display peptide library (1 × 10^10^ diversity, tenfold oversampled) at a 1:25 dilution in a 96-well, deep-well plate format. Antibody-bound bacterial clones were selected with 50 µl Protein A/G Sera-Mag SpeedBeads (GE Life Sciences, 17152104010350) (IgG). The selected bacterial pools were resuspended in growth medium and incubated at 37 °C with shaking overnight at 300 rpm to propagate the bacteria. Plasmid purification, PCR amplification of peptide-encoding DNA and barcoding with well-specific indices was performed as described. The samples were normalized to a final concentration of 4 nM for each pool and run on the Illumina NextSeq 500 system. Every 96-well plate of samples processed for this study contained healthy control run standards to assess and evaluate assay reproducibility and possible batch effects.

For IgM isotype screening by SERA, the above IgG protocol was adjusted as follows: (1) after serum incubation with the library, *Escherichia coli* cells were centrifuged, the supernatant was removed and the cells were resuspended in 500 µl 1× PBS containing a 1:100 dilution of biotin-SP (long-spacer) conjugated donkey anti-human IgM secondary antibodies (Jackson Immunoresearch, 709-066-073); (2) the plate was incubated for 1 h at 4 °C with orbital shaking (800 rpm), the cells were again centrifuged, the supernatant was removed and cells were resuspended in 700 µl of 1× PBS + 100 µL of Dynabeads MyOne streptavidin-T1-coated magnetic beads (Thermo Fisher Scientific, 65601); (3) the plate was incubated for 1 h at 4 °C with orbital shaking (800 rpm), after which time the plate was magnetized and the bead–antibody complex along with their bound peptide-bearing cells were captured. All of the subsequent steps were identical for IgG and IgM screening as described. IgM antibody repertoires were evaluated for EBV antibodies in two ways; (4) samples were analysed on an existing EBV IgM epitope panel that was developed and validated on clinically confirmed mononucleosis patients and EBV IgM negative controls.

#### PIWAS analysis

The published PIWAS method^65^ was used to identify antigen and epitope signals against the UniProt reference SARS-CoV-2 proteome (UP000464024). For each sample, approximately 1–3 million 12-mers were obtained from the SERA assay and these were decomposed into constituent 5- and 6-mers. An enrichment score for each *k*-mer was calculated by dividing the number of unique 12-mers containing the *k*-mer divided by the number of expected *k*-mer reads for the sample, based on amino acid proportions in the sample. A *z* score per *k*-mer was then calculated by comparing the enrichment score with those from a large pre-pandemic cohort (*n* = 1,500) on a log_10_ scale. A PIWAS value at each amino acid position along a protein sequence represents an averaged score within a 5-amino-acid frame using the tiling *z* scores of 5-mers and 6-mers spanning the sequence. 95th quantile bands were calculated on the basis of each population separately.

#### Protein-wide identification of epitopes

Protein-wide identification of epitopes methodology for epitope identification was performed to locate regions on a protein sequence that had stronger outlier signals in the case samples relative to control samples from a large pre-pandemic cohort (*n* = 1,500). The distribution of case sample values relative to the control was analysed at each amino acid position on the SARS-CoV-2 spike protein sequence. Specifically, at each position, all case and control sample values were normalized using median absolute deviation based on the distribution of the control sample values. An outlier threshold was defined as *Q*_75_ + 1.5 × (*Q*_75_ − *Q*_25_), where *Q*_*x*_ is the *x*th percentile of the control values at that specific position^66^. An outlier sum statistic was then calculated as the sum of signal values above the outlier threshold in the case samples^67^. A null distribution for the outlier sum value was calculated by permuting case/control labels and recalculating 1,000 times. A *P* value was calculated based on a *z* score by comparing the observed outlier sum statistic to the null distribution. A significant *P* value threshold was set to 0.001 after FDR adjustment by the Benjamini–Hochberg procedure and an outlier sum threshold was set to the 99.5th percentile value of all positions with FDR-adjusted *P* > 0.001. All sequence positions that exceeded both thresholds were identified, and adjacent positions were merged into regions representing epitopes on the protein.

#### IMUNE-based motif discovery

Peptide motifs representing epitopes or mimotopes of SARS CoV-2-specific antibodies were discovered using the IMUNE algorithm^68^. A total of 164 antibody repertoires from 98 hospitalized individuals from the Yale IMPACT study were used for motif discovery. The majority of individuals was confirmed to be SARS-CoV-2 positive by nucleic acid testing. IMUNE compared around 30 disease repertoires with about 30 pre-pandemic controls and identified peptide patterns that were statistically enriched (*P* ≤ 0.01) in ≥25% of disease and absent from 100% of controls. Multiple assessments were run with different subsets of cases and controls. Peptide patterns identified by IMUNE were clustered using a point accepted mutation 30 (PAM30) matrix and combined into motifs. The output of IMUNE included hundreds of candidate IgG and IgM motifs. A motif was classified as positive in a given sample if the enrichment was ≥3 times the s.d. above the mean of the training control set. The candidate motifs were further refined based on at least 98% specificity. The final set of motifs was validated for sensitivity and specificity on an additional 1,500 pre-pandemic controls and 406 unique confirmed COVID-19 cases from four separate cohorts.

#### Motif grouping by similarity

For SARS-CoV-2, motifs were grouped if they shared at least 3 of 5 amino acid identities, resulting in 76 motifs being assigned into 24 groups. The motif within an epitope group with the greatest sensitivity and mean enrichment was included in the SARS-CoV-2 Infection IgG panel results. In some cases, two motifs were selected from the same group as their combination improved sensitivity. The remaining motifs that did not fall into a group were further down-selected based on a specificity of >99.5%, leaving 24 additional motifs.

### REAP

#### REAP library expansion

The initial yeast library (Exo201) was generated as previously described^16,69^. In Exo201, only extracellular domains >49 amino acids in length were included in the library. To generate the library used for this study, Exo201 was created with all extracellular domains of multi-pass membrane proteins greater than 15 amino acids and 225 extracellular viral antigens. DNA for new antigens was synthesized as either a gene fragment (for antigens over 300 nucleotides) or as an Oligo pool by TWIST Bioscience, containing a 5′ sequence (CTGTTATTGCTAGCGTTTTAGCA) and 3′ sequence (GCGGCCGCTTCTGGTGGC) for PCR amplification. The oligo pool was PCR-amplified and transformed into yeast with barcode fragments, followed by barcode–antigen pairing identification as previously described^1,2^. This new yeast library was then pooled with the initial library (Exo201) at a ratio of 1:1 to generate the new version of the library (Exo205), which contained 6,452 unique antigens.

#### REAP protocol

Participant IgG isolation and REAP selections were performed as previously described^16,69^. In brief, IgG was purified from participant plasma using protein G magnetic beads followed by adsorption to yeast transformed with the pDD003 empty vector to remove yeast-reactive IgG. The Exo205 yeast library was induced in SGO-Ura medium, and 10^8^ induced yeast cells were washed with PBE and added to wells of a sterile 96-well plate. Then, 10 μg of purified participant IgG was added to the yeast library in duplicate in 100 μl PBE and incubated for 1 h at 4 °C. Yeast cells were washed with PBE and incubated with 1:100 biotin anti-human IgG Fc antibody (BioLegend, QA19A42) for 30 min. Yeast cells were washed with PBE and incubated with a 1:20 dilution of Streptavidin MicroBeads (Miltenyi Biotec, 130-048-101) for 30 min. Yeast were resuspended in PBE and IgG-bound yeast were isolated by positive magnetic selection using the MultiMACS M96 Separator (Miltenyi Biotec) according to the manufacturer’s instructions. Selected yeast were resuspended in 1 ml SDO −Ura and incubated at 30 °C for 24 h and then plasmid DNA was collected for NGS analysis. In brief, DNA was extracted from yeast libraries using Zymoprep-96 Yeast Plasmid Miniprep kits or Zymoprep Yeast Plasmid Miniprep II kits (Zymo Research, D2007) according to the standard manufacturer protocols. A first round of PCR was used to amplify a DNA sequence containing the protein display barcode on the yeast plasmid. A second round of PCR was performed on 1 µl of step 1 PCR product using Nextera i5 and i7 dual-index library primers (Illumina). PCR products were pooled, run on a 1% agarose gel and DNA corresponding to the band at 257 bp was cut. DNA (NGS library) was extracted using the QIAquick Gel Extraction Kit (Qiagen, 28704) according to standard manufacturer protocols. The NGS library was sequenced using the Illumina NextSeq 2000 system and an NextSeq 2000 P3 100 cycles sequencing kit (Illumina, 20040559) with 75 bp single-end sequencing according to standard manufacturer protocols. Approximately 500,000 reads (on average) per sample were collected and the preselection library was sampled at ten times greater read depth than the other samples. Samples with less than 50,000 reads were classified as a sequencing failure and removed from further analysis.

#### REAP data analysis

REAP scores were calculated as previously described^16,69^. In brief, barcode counts were extracted from raw NGS data using custom codes and counts from technical replicates were summed. Next, aggregate and clonal enrichment was calculated using edgeR^70^ and custom computer scripts. Aggregate enrichment is the log_2_-transformed fold change of all barcodes associated with a particular protein summed in the post-library relative to the pre-library, with zeroes in the place of negative fold changes. log_2_-transformed fold change values for clonal enrichment were calculated in an identical manner, but barcode counts across all unique barcodes associated with a given protein were not summed. Clonal enrichment for a given reactivity was defined as the fraction of clones out of total clones that were enriched (log_2_ fold change ≥ 2). Aggregate (*E*_a_) and clonal enrichment (*E*_c_) for a given protein, a scaling factor (*β*_*u*_) based on the number of unique yeast clones (yeast that have a unique DNA barcode) displaying a given protein, and a scaling factor (*β*_*f*_) based on the overall frequency of yeast in the library displaying a given protein were used as inputs to calculate the REAP score, which is defined as follows:

2 $${\rm{REAP}}\;{\rm{score}}={E}_{{\rm{a}}}\times {({E}_{{\rm{c}}})}^{2}\times {\beta }_{u}\times {\beta }_{f}.$$

*β*_*u*_ and *β*_*f*_ are logarithmic scaling factors that progressively penalize the REAP score of proteins with low numbers of unique barcodes or low frequencies in the library, and are described in detail in previous publications^16,69^_._

Antigens with an average REAP score of greater than 0.5 across all of the samples were defined as non-specific and were excluded from further analysis. Autoantibody reactivities were defined as antigens with a REAP score of greater than or equal to 1.

#### REAP antigen ELISA validation

Ninety-six-well MaxiSorp plates (Thermo Fisher Scientific, 442404) were coated with 200 ng per well of recombinant EBV p23 protein (ProSpec, ebv-274) in PBS and incubated overnight at 4 °C. The plates were dumped out and incubated with 3% Omniblock non-fat dry milk (American Bioanalytical, AB10109-00100) in PBS for 2 h at room temperature. The plates were washed three times with 200 μl wash buffer (PBS 0.05% Tween-20). The samples were diluted in 1% Omniblock non-fat dry milk in PBS and added to the plate to incubate 2 h at room temperature. The plates were washed six times with wash buffer. Goat anti-human IgG Fc HRP (Sigma-Aldrich, AP112P) diluted 1:10,000 in 1% Omniblock non-fat dry milk in PBS was added to the plates and incubated 1 h at room temperature. The plates were washed six times. The plates were developed with 100 μl of TMB Substrate Reagent Set (BD Biosciences, 555214) and the reaction was stopped after 5 min by the addition of 2 N sulfuric acid. The plates were then read at a wavelength of 450 nm.

### Machine learning

#### Data preprocessing

All collected data for immune profiling were collated. Features containing redundant information were manually removed from the dataset (for example, nested flow cytometry populations include only the extant population).

All features were linearly scaled to unit variance and zero-centred using the R programming language base libraries^71,72^. The median absolute deviation was calculated for each feature across all samples, with missing values removed. Features with a median absolute deviation equal to zero or features where data were not available in at least half the samples were not included in downstream analysis. Before visualization of the data using PCA, features were also quantile-normalized using the ‘normalize.quantiles’ function of the preprocessCore package in R^73^.

#### Gale–Shapley matching of participants by demographics

To ensure that immunological features of participants in the LC cohort would be compared against the most similar set of controls in the CC and HC cohorts, the Gale–Shapley matching procedure was used^74^. The participants in the LC cohort were first matched against participants in the CC cohort. Unmatched participants in the LC cohort were subsequently matched against participants in the HC cohort. Preference lists required by the Gale–Shapley algorithm were determined using an affinity function calculated as the cosine similarity of participants in a unit-scaled and zero-centred demographics matrix containing age, sex, vaccination status and days from the initial onset of acute COVID-19. The matching was performed using the galeShapley.marriageMarket function of the matchingR package in R^71^. To evaluate matching efficacy, differences between groups in age, sex, vaccination status, acute COVID-19 hospitalization status and days from initial onset of acute COVID-19 were assessed using a *χ*^2^ test. For age, participants were segmented into groups as either less than 32 years of age, between 33 and 51 years of age, or greater than 52 years of age. For days from symptom onset, the participants were segmented into groups as either 1–2 months from acute infection, 2–5 months from acute infection, 6–8 months from acute infection or ≥9 months from acute infection. An *α* of 0.05 was used throughout.

#### Unsupervised analysis

PCA was performed on the set of normalized features for all of the matched participants^75^. To assess how well participants were grouped by all features, a *k*-NN classifier with *k* = 10 was applied separating participants with LC from those without (either convalescent participants or healthy controls). A *k* of 10 was chosen by heuristic as approximately equal to the square root of the number of samples included^76^. A range of values for *k* from 5 to 15 were tested and found to give similar results. Area under the receiver operating characteristic curve and 95% confidence intervals were calculated using DeLong’s method; *P* values were calculated using the Mann–Whitney *U* statistic^77,78^.

#### Supervised analysis

Principal component regression was applied to each of a predefined set of data segments: autoantibodies, SARS-CoV-2 antibodies, non-SARS-CoV-2 viral antibodies, plasma proteomics and flow cytometry readouts. The precise definitions of these data segments are provided as metadata. The first *n* principal components based on explained variance (see below for selection method) were selected from the normalized feature set and used to fit a logistic regression model (implemented as a binomial generalized linear regression with a logit link) for classification of participants with LC as compared to matched convalescent participants without long-term symptoms and uninfected controls.

To determine the optimal value for *n* (the number of principal components), values were scanned, and sevenfold cross validation was performed on the dataset. The average mean squared error was calculated for each cross-validation iteration at a particular value of *n*. For the binomial regression run using a logit link function, McFadden’s pseudo-*R*^2^ was calculated and averaged across each of the cross-validation folds.

Plots of explained variance and mean squared error across all scanned values for *n* were generated and visually inspected to choose an optimal value for *n* that maximized explained variance while minimizing overfitting as identified by increasing average mean squared error. This procedure was performed on each of the segments, and an optimal *n* was chosen for each of the following: autoantibodies (*n* = 5), SARS-CoV-2 antibodies (*n* = 3), non-SARS-CoV-2 viral antibodies (*n* = 32), plasma proteomics (*n* = 18) and flow cytometry (*n* = 21).

A model fitted on the first *n* principal components (or any linear transformation) was related to each of the original features as follows. Each principal component may be considered as a weighted linear combination of the original features. The principal component loading vectors were used to project the fitted beta values from the logistic regression model using the linearity of expectation, *E*(*X* + *Y*) = *E*(*X*) + *E*(*Y*), such that the estimated parameter for each variable was the weighted sum of the parameter estimates for the principal components to which it contributed. The variance of fit for each of the original features was similarly projected from the fitted principal components as the variance of a sum of random variables Var(*X* + *Y*) = Var(*X*) + Var(*Y*) + 2Cov(*X*, *Y*). *P* values were calculated for each variable in the original feature space using *z* scores.

After per-segment model construction and evaluation, features with a Bonferroni-corrected *P* value of less than 0.05 were selected for inclusion in a final principal component regression. These selected features were considered as a separate integrated data segment and were processed in the same way as each individual data segment with a selected (*n* = 6) number of included principal components. A LASSO regression was used to select a subset of the features with *P* values less than 0.05 as a minimal model, and McFadden’s pseudo-*R*^2^ was calculated.

An implementation has been made publicly accessible as an R library at GitHub (https://github.com/rahuldhodapkar/puddlr).

#### Symptom bi-clustering

Participants with LC were clustered on the basis of binary self-reporting of LC symptoms. Hamming distance was used with complete linkage clustering as an agglomeration method. Visualization of the bi-clustering was performed using the ComplexHeatmap package in R^79^. Cluster stability was assessed by bootstrapped resampling with 100 iterations using the fpc package in R^80^.

### General statistical analysis

Study sample sizes were not predetermined through formal power analysis. Study investigators were not blinded to participant status. Specific statistical methodology can be found in relevant figure legends and manuscript text. Generally, comparison of immunophenotypic features including systemic cytokine levels and antibody concentrations between study cohorts was performed using estimates of group medians, primarily with nonparametric Kruskal–Wallis tests. All statistical tests were two sided.

The difference in median between the days from the symptom onset of acute COVID-19 in the LC and CC groups was assessed using a two-tailed Brown–Mood median test with an *α* of 0.05. The test was performed using the coin package in R^81^. Flow cytometry populations were assessed using estimates of group means with permutational testing using PERMANOVA to control for within-group heterogeneity (described above).

In cases in which Kruskal–Wallis testing indicated significant differences, post hoc testing using Dunn’s test was performed. Correction for multiple comparisons was performed using the Bonferroni or Bonferroni–Holm method as indicated. All statistical tests were performed using R, PRISM and MATLAB.

### Reporting summary

Further information on research design is available in the Nature Portfolio Reporting Summary linked to this article.

## Online content

Any methods, additional references, Nature Portfolio reporting summaries, source data, extended data, supplementary information, acknowledgements, peer review information; details of author contributions and competing interests; and statements of data and code availability are available at 10.1038/s41586-023-06651-y.

### Supplementary information

Reporting Summary

Supplementary Table 1 Antibody clones and dilutions used for flow cytometry analysis. Excel file containing a list of antibodies used in flow cytometry analysis.

Supplementary Table 2 Viral antigens included in REAP analysis. Excel file containing a list of viral antigens used in REAP analysis.

Supplementary Table 3 MY-LC clinical and immunological data. Excel file containing various immunological and clinical data used for analyses throughout the manuscript.

Supplementary Table 4 EXT-LC clinical and immunological data. Excel file containing various immunological and clinical data from the external LC group used for analyses throughout the manuscript.

## Figures and Tables

**Extended Data Fig. 10:**
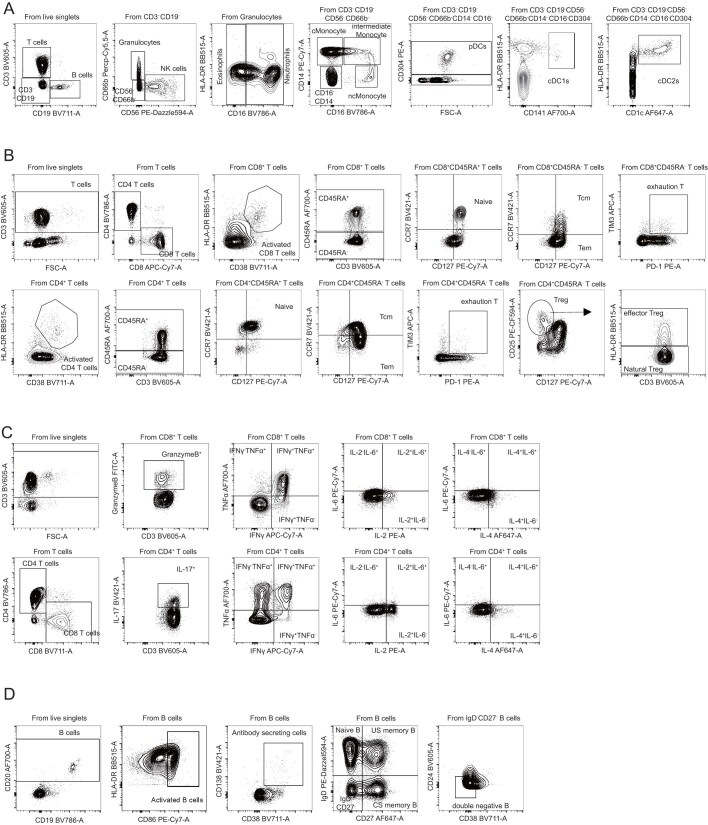
Flow Cytometry gating schematics. (**A**–**D**). Various gating strategies for granulocyte and myeloid populations (**A**), T lymphocytes (**B**), intracellular cytokine staining (**C**), and B lymphocytes (**D**).

**Extended Data Table 1:**
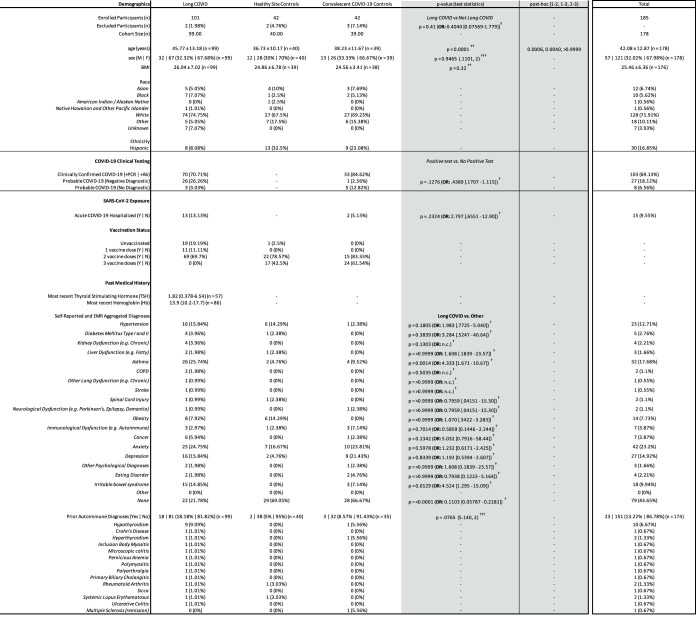
Clinical Demographics of MY-LC Cohort

Summary demographic and clinical characteristics for the MY-LC Study. Participants were stratified into three study arms at enrollment: (1) Long COVID (prior SARS-CoV-2 infection with persistent, unexplained symptoms); (2) healthy study site cohort (no prior SARS-CoV-2 infection); or (3) convalescent COVID-19 cohort (prior SARS-CoV-2 infection without persistent symptoms). Various demographic features and clinical characteristics are reported by row for each cohort (row measurement units are specified in parentheses). Within each cell, counts or clinical feature averages are reported, with sample standard deviations, relative cohort percentages, and participant numbers reported where pertinent. Results from statistical tests are reported as p-values and accompanying test statistics: † Chi-square test p-value (Chi-square test statistic, degrees of freedom (df)); †† Kruskal-Wallis ANOVA p-value; ††† Fisher’s exact test p-value (Odd’s Ratio: [95% Confidence Interval (Baptista-Pike)]); ‡ Mann-Whitney U test p-value. Post-hoc comparisons were conducted using Dunn’s test with Tukey’s correction for multiple comparison (column comparison order left-right: 1-2, 1-3, 2-3). Participant medical histories were collected and collated from binary self-reports of prior medical history and review of electronic medical records by study staff (positive responses in either participant self-report or EMR review were considered an overall binary positive response). Abbreviations: *n, number; M, male; F, female; BMI, body mass index; +PCR, positive result from SARS-CoV-2 nucleic acid test; +Ab, positive result from SARS-CoV-2 antibody test; Y, Yes; N, No*.

**Extended Data Table 2:**
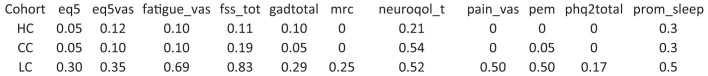
Normalized survey responses across MY-LC cohorts

Survey responses for participants are organized by individual instruments (columns) and MY-LC cohorts (rows). Participant responses for each survey instrument were summed and normalized using standard min-max normalization procedures such that a value of 1 equals the maximum possible aggregate score and 0 equals the minimum possible aggregate score. Additionally, individual survey elements were oriented through inversion such that higher normalized scores on each instrument indicate a higher intensity or degree of agreement with survey prompts. For each cohort, median values are displayed.

**Extended Data Table 3:**
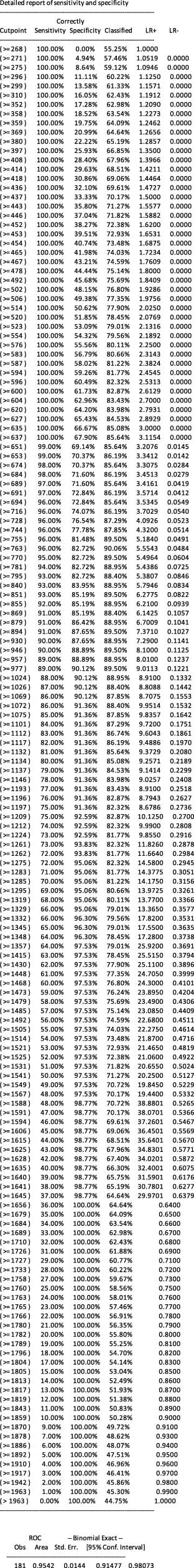
Determinations of optimal LCPS threshold

Classification metrics across different LCPS thresholds (‘Cut-offs’) (*Upper table*). Summary area-under the curve (AUC) statistics and bootstrap confidence intervals for Receiver-Operator curve analysis (ROC) (*lower table*).

**Extended Data Table 4:**
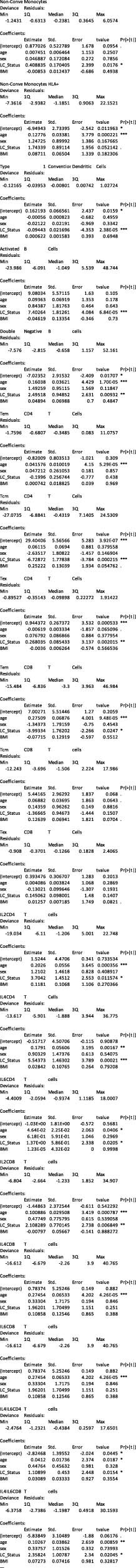
Modeling of select flow cytometry populations

(**A**–**L**) Detailed linear modeling results are reported for various cytokine producing T cell populations analyzed by flow cytometry.

**Extended Data Table 5:**
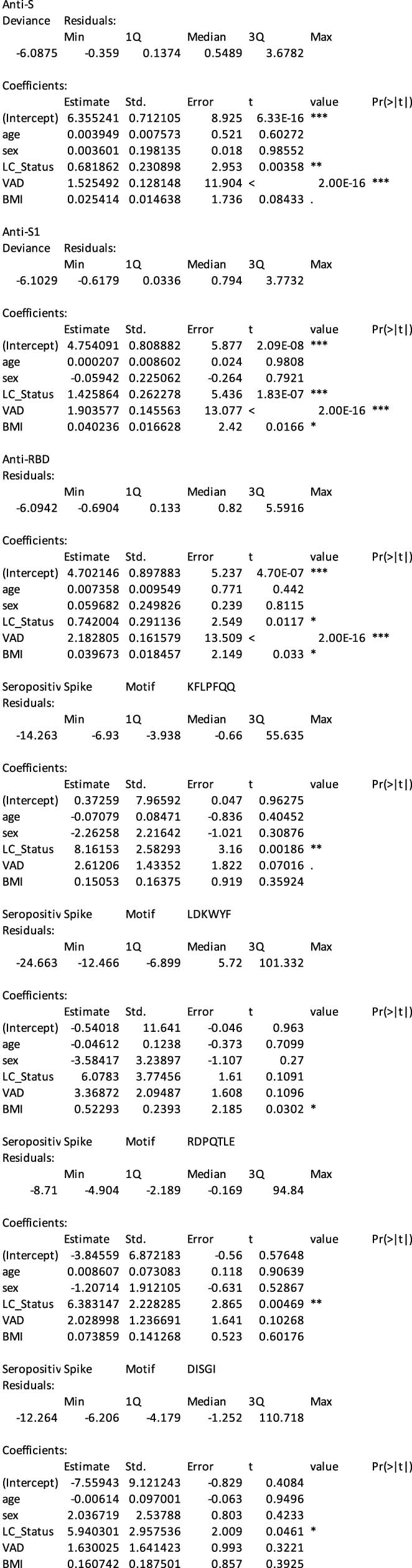
Modeling of anti-SARS-CoV-2 antibody and linear motif responses

(**A**–**E**) Detailed linear modeling results are reported for SARS-CoV-2 specific antibody responses and peptide motifs with corresponding model formulations.

**Extended Data Table 6:**
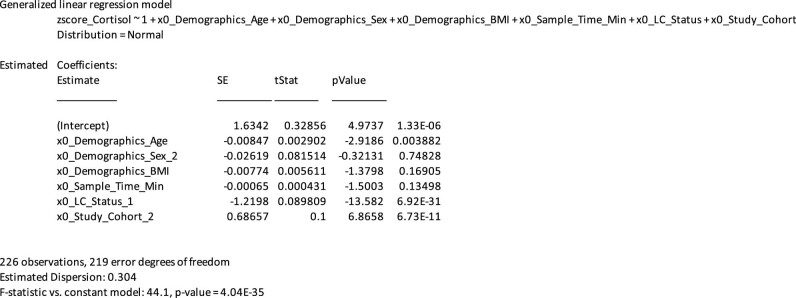
Modeling of cortisol levels

Detailed linear modeling results are reported for cortisol levels across groups with corresponding model formulation.

**Extended Data Table 7:**
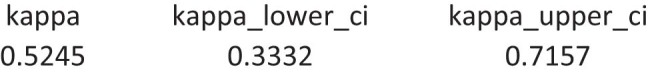
Inter-model Long COVID classification comparison

Cohen’s Kappa analysis of agreement between LCPS and Integrated immunological classification of Long COVID status.

## Data Availability

All of the raw .fcs files for the flow cytometry analysis are available at the FlowRepository platform (http://flowrepository.org/) under repository ID FR-FCM-Z6KL. Protein structures were visualized using the PDB repository under the following accession numbers: trimeric spike (PDB: 6VXX) and EBV gH/gL (PDB: 5T1D). Raw data are included in Supplementary Tables 3, 4.

## Code availability

Computer codes are available as indicated (https://github.com/rahuldhodapkar/puddlr) or are otherwise available on request.
